# A single species, different repeatomes? Genomic plasticity in *Passiflora foetida* L. complex and comparative insights across the subgenus *Passiflora*

**DOI:** 10.1007/s10577-026-09804-7

**Published:** 2026-08-03

**Authors:** Arthur Monteiro, Mariela A. Sader, Jéssica Nascimento, Gustavo Luna, Daniela Cristina Imig, Andrea Pedrosa-Harand

**Affiliations:** 1 Laboratory of Plant Cytogenetics and Evolution, Department of Botany, Biosciences Centre, Federal University of Pernambuco, Recife, PE 50670‑901 Brazil; 2 Instituto Multidisciplinario de Biología Vegetal, (Consejo Nacional de Investigaciones Científicas y Técnicas - Universidad Nacional de Córdoba), C.C. 495, Córdoba, Argentina; 3 Laboratory of Plant Cytogenetics and Evolution, DEDC, Campus VIII, State University of Bahia, Paulo Afonso, BA Brazil; 4 Herrero College, Curitiba, Paraná Brazil

**Keywords:** Centromere, Comparative genomics, Intraspecific variation, Retrotransposons, Satellite DNA

## Abstract

The genus *Passiflora* L. comprises approximately 650 species, and exhibits high diversity in the Neotropics, especially the subgenus *Passiflora*. Several species of this subgenus have an abundant repetitive fraction, consistent with their increased genome sizes. However, the repetitive DNA fraction of *Passiflora foetida* (*n* = 10), sister to the other species of the subgenus (*n* = 9) and the species with the smallest genome, has not yet been described in detail. In addition, this species comprises a complex of morphological varieties with a wide geographical distribution. The aim of this study was to characterize its repeatome variability in comparison to other species in section *Dysosmia* and other sections in the same subgenus, and to investigate whether it may reflect the high variability reported for this species complex. The results showed that *P. foetida* has a proportion of repetitive DNA fraction in part consistent with its small genome size, but with marked intraspecific variation. The LTR Ty1/copia retrotransposons were the most abundant, as in the other species of the subgenus, but Ty3/gypsy represented a much smaller fraction compared to the others. The variation observed within *P. foetida* was greater than that detected among *Dysosmia* species. Satellite DNAs were partly unique and partly similar to species from different subgenera, supporting its phylogenetic position. In addition, one *Dysosmia*-specific satellite showed a proximal chromosomal location, making it the first candidate of a centromeric repeat for the genus, despite its variation in abundance among chromosomes.

**Supplementary Information:**

The online version contains supplementary material available at 10.1007/s10577-026-09804-7.

## Introduction

Angiosperms exhibit extensive genomic variation, including differences in DNA content, chromosome number, and both structural and compositional genome features (Jiao et al. 2011; Carta et al. 2020; Carta et al. 2022). A substantial portion of this variation is associated with repetitive DNA, which constitutes a major fraction of plant genomes and plays a central role in chromosome organization and evolution (Heslop-Harrison and Schwarzacher 2011; Jiang 2019). Repetitive elements, such as retrotransposons and satellite DNAs, collectively referred to as the repeatome, can influence genome size, chromosomal architecture, and evolutionary dynamics, contributing to genomic differentiation among lineages (Bennett and Leitch 2005; Bourque et al. 2018).

Repeatome variation and its impacts have been widely investigated at the interspecific level, reflecting processes such as geographic isolation, local adaptation, and demographic history (Ricci et al. 2018; Castro et al. 2024). However, studies focusing on intraspecific variation remain scarce. Repetitive DNA can evolve rapidly and accumulating evidence suggests that it varies even among individuals of the same species (Kang et al. 2023; Lian et al. 2024; Cheng et al. 2025). In this context, characterizing intraspecific repeatome variation may provide important insights into the mechanisms underlying genomic diversity and its role in recent evolutionary processes, such as speciation.

The genus *Passiflora* L. includes over 650 species (MacDougal and Tillet 2022; Kuethe 2024), predominantly distributed throughout the Neotropics, with additional occurrences in other regions of the Americas, Africa, and Asia. It represents the largest genus within the family Passifloraceae Juss. ex Roussel (Cervi and Imig 2013; POWO, 2025), and is currently divided into six main subgenera: *Astrophea* (DC.) Mast., *Decaloba* (DC.) Rchb., *Deidamioides* (Harms) Killip, *Passiflora* Feuillet & MacDougal (Feuillet and MacDougal 2003), *Tetrapathea* (DC.) P. S. Green, and *Tryphostemmatoides* (Harms) Killip (Ulmer and MacDougal 2004; Krosnick et al. 2009; 2013; Buitrago et al. 2018; MacDougal and Tillet 2022).

The subgenus *Passiflora* comprises approximately 250 species and is primarily characterized by large flowers bearing multiple series of corona filaments. It is the most diverse subgenus in South America and the most widely recognized, largely due to the economic importance of cultivated species such as sour passion fruit (*Passiflora edulis* Sims) and sweet passion fruit (*Passiflora alata* Curtis) (Ulmer and MacDougal 2004). From a cytogenetic perspective, the subgenus *Passiflora* is characterized by a basic chromosome number of* x* = 9 (Hansen et al. 2006; Sader et al. 2019), with the notable exception of *Passiflora foetida* L. and closely related species from section *Dysosmia*, which presents *n* = 10 (Zirpoli et al. 2025). Phylogenetic studies place *P. foetida* as the earliest-diverging lineage and sister to all other species in the subgenus (Krosnick et al. 2013; Cauz-Santos et al. 2020). Considering an ancestral chromosome number of *x* = 12 for the genus (Melo and Guerra 2021), the *n* = 10 karyotype of *P. foetida* may represent an intermediate state in a descending dysploid series leading to *n* = 9.

Previous studies in *Passiflora* have shown that genome size variation is correlated to flower size (Yotoko et al. 2011) and strongly associated with the dynamics of repetitive elements, particularly LTR-retrotransposons from the Ty1/copia and Ty3/gypsy lineages (Sader et al. 2021). Despite this, current knowledge of repeatome composition and organization is still limited to a small number of *Passiflora* species (Sader et al. 2021). Even in species with chromosome-level genome assemblies, such as *P. edulis*, key aspects of repetitive DNA organization, including the identification of centromeric sequences, remain poorly understood (Ma et al. 2021; Xia et al. 2021). More recently, the chromosome-level assembly of *P. foetida* revealed a predominance of Ty1/copia elements and a low abundance of Ty3/gypsy (Zou et al. 2023).

*Passiflora foetida* is a widely distributed climbing vine native to warm regions of the Americas and the Caribbean. However, it has become naturalized and, in some regions, invasive in other parts of the world, including Africa, South Asia, Australia and Hawaii (Hopley et al. 2021; 2026; Zou et al. 2023). Phylogeographic studies indicated a complex history of introduction and dispersal, with evidence of genetic structure among populations (Hopley et al. 2021, 2026). In addition, the species exhibits marked floral and vegetative morphological variation with flower colours ranging from purplish to white, which complicates classifications due to extensive character overlap (Svoboda 2023). This variability has resulted in the application of multiple names to infraspecific taxa (Killip, 1938; Vanderplank 2013; Svoboda et al. 2016). Following Vanderplank (2013), six varieties are currently recognized: *P. foetida* var. *foetida* L., var. *nigelliflora* (Hooker), var. *oaxacana* Killip, var. *acapulsensis* Killip, var. *ellisonii* Vanderplank, and var. *baraquiniana* (Lem.) Vanderplank (Vanderplank 2013). Despite this phenotypic diversity and plasticity, the relationships among these varieties remain poorly resolved. In this context, investigating the composition and organization of the repetitive fraction across different varieties of *P. foetida* and closely related species may help elucidate patterns of genomic variation at the intraspecific level and their relationship to diversification within the species. Furthermore, repeatome analysis may provide informative markers for distinguishing lineages and contribute to resolving species complexes.

The aim of this study was to investigate the composition and organization of the repetitive fraction in the genome of different varieties of *P. foetida* (*n* = 10), thereby contributing to a better understanding of the evolutionary mechanisms shaping the repeatome in *Passiflora* and its role in genomic diversity. The usefulness of repeat composition for resolving phylogenetic relationships at intraspecific level and thus for contributing to elucidate species complexes was also addressed. Specifically, we addressed the following questions: (i) What does the repetitive fraction of *P. foetida* reveal about repeatome evolution within the subgenus *Passiflora*? (ii) Is there intraspecific variation in the repeatomes of *P. foetida*? (iii) Which repetitive elements are associated with potential genomic differentiation among the species’ varieties? (iv) Are there specific patterns in the organization of the repetitive fraction that support the distinction among varieties? (v) Is there intraspecific variation in the chromosomal distribution of the main satellite DNA sequences identified?

## Methodology

### Plant material

**Fig. 1:**
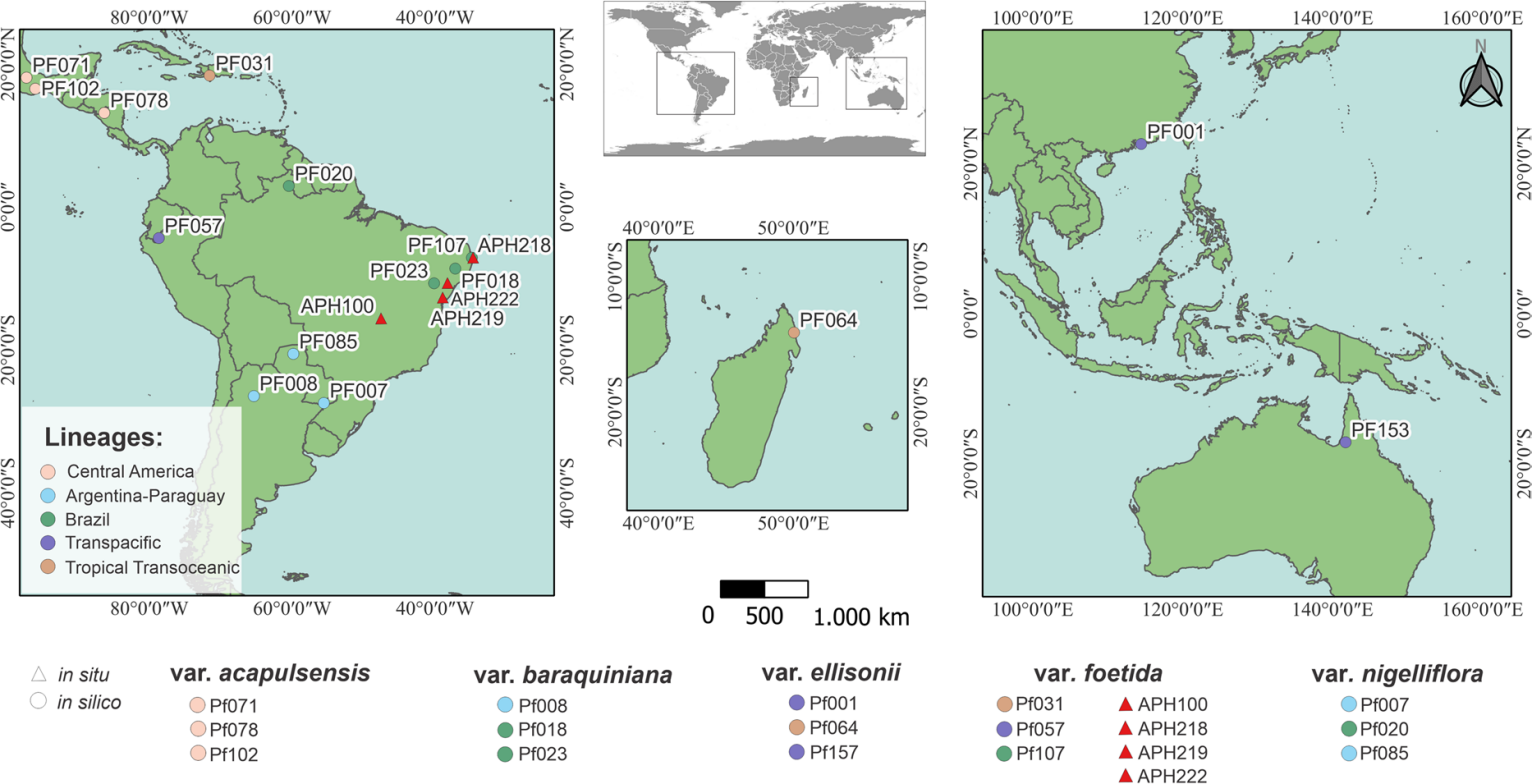
Geographic distribution of the analysed *P. foetida* accessions in relation to their phylogenetic clades (Hopley et al. 2021) and taxonomic identification (present work). Accessions represented by circles were used for in silico analyses, whereas those represented by triangles were collected in this study and used for in situ analyses

Two accessions of *Passiflora foetida* were obtained for cytogenetic analyses through collaboration with *Passiflora* Germplasm Banks at Embrapa Cerrado—APH100 (Planaltina, Federal District, Brazil) and Embrapa Cassava and Tropical Fruits—APH219 (Cruz das Almas, Bahia, Brazil). Additional two accessions (APH218 and APH222) were collected from different states of Northeast Brazil (Fig. 1, Table S1). Plants derived from germinated seeds were cultivated in the Experimental Garden of the Laboratory of Cytogenetics and Plant Evolution, Department of Botany, Federal University of Pernambuco (UFPE). Low-coverage Illumina sequence data for herbarium accessions of *P. foetida* were retrieved from the European Nucleotide Archive (ENA; PRJEB43378, Hopley et al. 2021), and genome assembly, as well as short reads were obtained from the Genome Sequence Archive for the Chinese accession here referred to as PF1 (GSA; PRJCA020083, Zou et al. 2023). Geographic maps were generated using QGIS 3.34.1.

### Data processing, clustering analysis, and satellite DNA characterization

A subset of sequences from *P. foetida* accessions and closely related species were obtained from Hopley et al. (2021; Table S2). The accessions used for characterization of its repetitive fraction were chosen to represent its molecular and taxonomic diversity, as well as its wide geographic distribution (Fig. 1, Table S2). Taxonomic identifications were performed according to Vanderplank (2013) using vouchers associated with each accession deposited at the Missouri Botanical Garden (USA) or inferred from the documented occurrence of specific varieties. In total, 15 accessions were selected, comprising three representatives of each identified variety (Fig. 1, Table S2). Additionally, sequence data for the individual PF1, used for genome assembly (PRJCA020083; Zou et al. 2023), were obtained from the Genome Sequence Archive (GSA).

For interspecific comparative analyses, one accession of *P. foetida* (PH18) was compared to publicly available sequences from *P. caerulea*, *P. coccinea*, *P. edulis*, *P. incarnata*, *P. ligularis*, *P. quadrangularis*, and *P. suberosa* (PRJEB43378, Hopley et al. 2021), as well as from *P. cincinnata* and *P. organensis* (Sader et al. 2021). This accession was chosen for individual and comparative analyses with other species because of its close geographical proximity to the Brazilian accessions collected for cytogenetic analyses. Two further accessions, representing the highest and lower abundances of repeats, were selected and also analysed individually for validating the results obtained in the comparative analysis. Comparison among species of the section *Dysosmia* was based on available data for *P. ciliata* (PH64) and *P. vesicaria* (PH103; PRJEB43378, Hopley et al. 2021), as some accessions initially identified as *P. foetida senso lato* were identified here as belonging to these species. For all species with genome sizes available, the size of the reads (150 bp) were considered for achieving 0.1 × genome coverage for repetitive DNA characterization. In the individual analyses of *P. foetida*, for instance, 293,400 reads were used, achieving 0.1 × genome coverage. Despite this standardization, the effective coverage reported in the output of the comparative analyses was lower (e.g., ~ 0.03 ×), reflecting the subsampling strategy automatically applied by the RepeatExplorer pipeline in this case. Because the genome size of *P. ciliata* is unknown, the same number of reads (1,000,000, trimmed to 100 bp) was used for all three species of section *Dysosmia* in a separate analysis, and genome coverage was calculated only for the two species with genome size estimates available. Two species from subgenus *Decaloba* were used as outgroups (Fig. S1, Table S3).

All sequences were quality-filtered using a cutoff value of Q ≥ 20, requiring at least 90% of bases to meet or exceed this threshold, using the FASTX-Toolkit (Gordon and Hannon 2010; http://hannonlab.cshl.edu/fastx_toolkit/) implemented in RepeatExplorer2. Similarity-based clustering was performed using the RepeatExplorer2 pipeline on the ELIXIR-CERIT Galaxy server (https://www.repeatexplorer-elixir.cerit-sc.cz), applying default parameters (Novák et al. 2020). For comparative analyses, reads from the accessions were labelled with unique prefixes, concatenated, and analysed jointly. Final genome coverage, after automatic sampling by RepeatExplorer2 and removal of mitochondrial and plastid reads, was calculated as: coverage = (r × l)/g, where *r* is the number of reads analysed after clustering, *l* is the read length, and *g* is the haploid genome size in base pairs.

For characterization of the repetitive fraction, clusters representing at least 0.01% abundance were automatically annotated and manually curated following the nomenclature proposed by Neumann et al. (2019). A custom database of *Passiflora* satellite DNA sequences (Sader et al. 2021) was included in both individual and comparative analyses. Unclassified clusters were further analysed by similarity searches using BLASTN against the non-redundant protein sequence database in public repositories (https://blast.ncbi.nlm.nih.gov/Blast.cgi). The proportion of each repetitive sequence was calculated as the number of reads assigned to each cluster relative to the total number of reads in the final dataset, excluding plastid and mitochondrial sequences.

The dataset used for repeatome characterization of all species and *P. foetida* accessions were also analysed using the TAREAN tool implemented in RepeatExplorer2. TAREAN identifies tandemly organized repetitive DNAs and reconstructs putative satellite DNA (satDNA) consensus sequences based on *k-mer* analysis (Novák et al. 2020). Tandem organization was further assessed using dot-plot analyses with default parameters in Geneious Prime v.7.0.6. Dot plot analyses among all satellite DNA sequences simultaneously were performed in Dotter (Sonnhammer and Durbin 1995) through command-line execution in a Linux environment, allowing the visualization of sequence similarity among the analysed sequences. Homology among satellite clusters identified across species was assessed following the classification criteria proposed by Ruiz-Ruano et al. (2016), with minor modifications: monomers sharing 50–80% identity across their entire length were considered different families within the same superfamily; those with 80–95% identity were treated as distinct subfamilies within a family; and sequences with ≥ 95% identity were classified as monomeric variants. For this, we used the Global alignment with free end gaps algorithm in Geneious v.7.0.6.

For the *in silico* analysis of the repetitive fraction in the assembled genome, repeat annotation was performed using CARP (Comprehensive Annotation of Repeats Pipeline; Novak 2025), following the developer’s official protocol available at the GitHub repository (https://github.com/kavonrtep/CARP). This automated Snakemake-based workflow identifies, classifies, and quantifies repetitive sequences in assembled genomes. The pipeline integrates the DANTE (Novák et al. 2024) toolkit for the annotation and classification of transposable elements, and TideCluster (https://github.com/kavonrtep/TideCluster) for the identification and characterization of tandem repeats, enabling comprehensive detection of both dispersed and tandem repetitive elements. This framework ensures standardized, reproducible, and comprehensive repeat annotation in genome assemblies.

All analyses were conducted in accordance with the guidelines provided in the official repository, ensuring consistency across preprocessing, repeat identification, and classification steps. For the visualization of the distribution of tandem repeats in the assembled pseudochromosomes (Zou et al. 2023), manually consensus sequences were also mapped against the genome using Geneious (v. 9.1.8). Subsequently, the results were processed with bedtools v.2.31.0 (Linux) to calculate sequence density within successive 1 kbp sliding windows along the pseudochromosomes. The distribution across the assembled chromosomes was plotted using ShinyCircos (Yu et al. 2018).

### Slide preparation

Root tips from potted plants and germinated seeds grown on Petri dishes were collected and treated with 2 mM 8-hydroxyquinoline (8-HQ) for 4.5 h at 10 °C. Samples were then fixed in Carnoy’s solution (ethanol:glacial acetic acid, 3:1) for at least 1 h at room temperature and stored at - 20 °C. For slide preparation, roots were washed twice in distilled water and digested at 37 °C in a solution containing 2% cellulase Onozuka (Serva) and 20% pectinase (Sigma) (v/v) for 1 h. Preparations followed a modified air-drying protocol (Ribeiro et al. 2017a, b). Slides were stained with 1 μg/mL 4',6-diamidino-2-phenylindole (DAPI) in a mounting medium (glycerol:McIlvaine buffer pH 7.0, 1:1; v/v) and visualized under a fluorescence microscope to select the best preparations. Selected slides were destained in Carnoy's fixative for 30 min and in absolute ethanol for 1 h and then stored at - 20 °C until further use.

### Fluorescence *in situ* hybridization (FISH)

Fluorescence *in situ* hybridization (FISH) experiments were performed following the protocol described by Pedrosa et al. (2002), using 5S rDNA probes (four oligomers based on conserved angiosperm sequences, pre-labelled at 3’ with Cy3 – Pre-Labelled Oligomer Probes, PLOPs – synthesized by Macrogen; Waminal et al. 2018) and 35S rDNA (clone p*Ta*71; Gerlach & Bedbrook, 1979), as well as the satellite DNAs Pqu01-100 and PquSat06-1083 (Sader et al. 2021). Except for the PLOPs, the remaining probes were labelled via nick translation using Cy3-dUTP, and Alexa488-dUTP was used for the p*Ta*71 probe.

Additionally, the most abundant satellite DNA sequence (PfoSat01-27) was synthesized as a pre-labelled oligonucleotide probe (Macrogen) with a Cy3 molecule at the 3’ end and hybridized using the non-denaturing FISH (ND-FISH) protocol adapted from Cuadrado et al. (2009). A hybridization solution (30 µL) containing 1 pmol (25 ng) of probe in 2 × SSC was applied, slides were covered with plastic coverslips and incubated in a humid chamber at room temperature for 2 h. After incubation, coverslips were removed in 2 × SSC, slides were washed in 4 × SSC/0.2% Tween20 at RT for 10 min and mounted in a DAPI-containing mounting medium (glycerol:McIlvaine buffer, pH 7.0, 1:1; v/v). FISH images were captured using a Leica DM5500B epifluorescence microscope equipped with a DFC 340FX digital video camera and the AF6000 software (Leica). The best-quality metaphase spreads were selected, captured images for each filter were superimposed using the lighten modus for the signal layers, pseudo coloured using the image adjustment Hue/Saturation function, and uniformly processed for brightness and contrast using Adobe Photoshop® (v.23.1.1).

### Phylogenetic analysis

Plastome sequences from all 15 accessions of *P. foetida* and 11 additional species were assembled through reference-based mapping using Geneious v.6.0.3 (Kearse et al. 2012), with the complete plastome of *Passiflora foetida* (NC_043825.1) serving as the reference. Reads were mapped using the default parameters of the "Map to Reference" function, and the resulting consensus sequences were aligned with MAFFT (Katoh and Standley 2013). For the intraspecific phylogenetic analyses, six complete plastomes representing species from *Passiflora* genus were included as outgroups: *P. oerstedii* Mast (NC_038124.1), *P. menispermifolia* Kunth (NC_043826.1), *P. retipetala* Mast (NC_038188.1), *P. mucronata* Lam (NC_053312.1), *P. edmundoi* Sacco (NC_053309.1), and *P. watsoniana* Mast (NC_053315.1). For the interspecific plastome phylogenetic analysis, species from section *Decaloba* were included, specifically *P. organensis* Gardner and *P. suberosa* L. In addition to the intraspecific plastome tree, a nuclear topology was also inferred using *P. foetida* rDNA cistron (MH768301.1) as the reference, with *P. organensis* designated as outgroup.

Interspecific phylogenetic inference was performed using FastTree v.2.1.11 (Price et al. 2009), implemented as a plugin in Geneious v.6.0.3 (Kearse et al. 2012), with default settings. Phylogenetic inference among accessions was conducted within a Bayesian framework using BEAST v.10.5.0 (Suchard et al. 2018). The final alignment contained less than 20% missing data. Two independent Markov chain Monte Carlo (MCMC) analyses were run for 50 million generations each, sampling every 5,000 generations. A GTR substitution model with four gamma-distributed rate categories, a Yule speciation prior, and an uncorrelated relaxed molecular clock were applied (Drummond et al. 2006). After discarding the first 10% of samples as burn-in, convergence and effective sample sizes (ESS) were assessed using Tracer v.1.7.2 (Rambaut et al. 2018). A maximum clade credibility tree was generated with TreeAnnotator (BEAST package), and node support was evaluated using Bayesian posterior probabilities. The calibration point for the plastome dated tree was sourced from TimeTree (Kumar et al. 2017), which references a 32-million-year-old origin for *Passiflora.*

## Results

### Comparative analysis among accessions and varieties of *P. foetida*

**Fig. 2:**
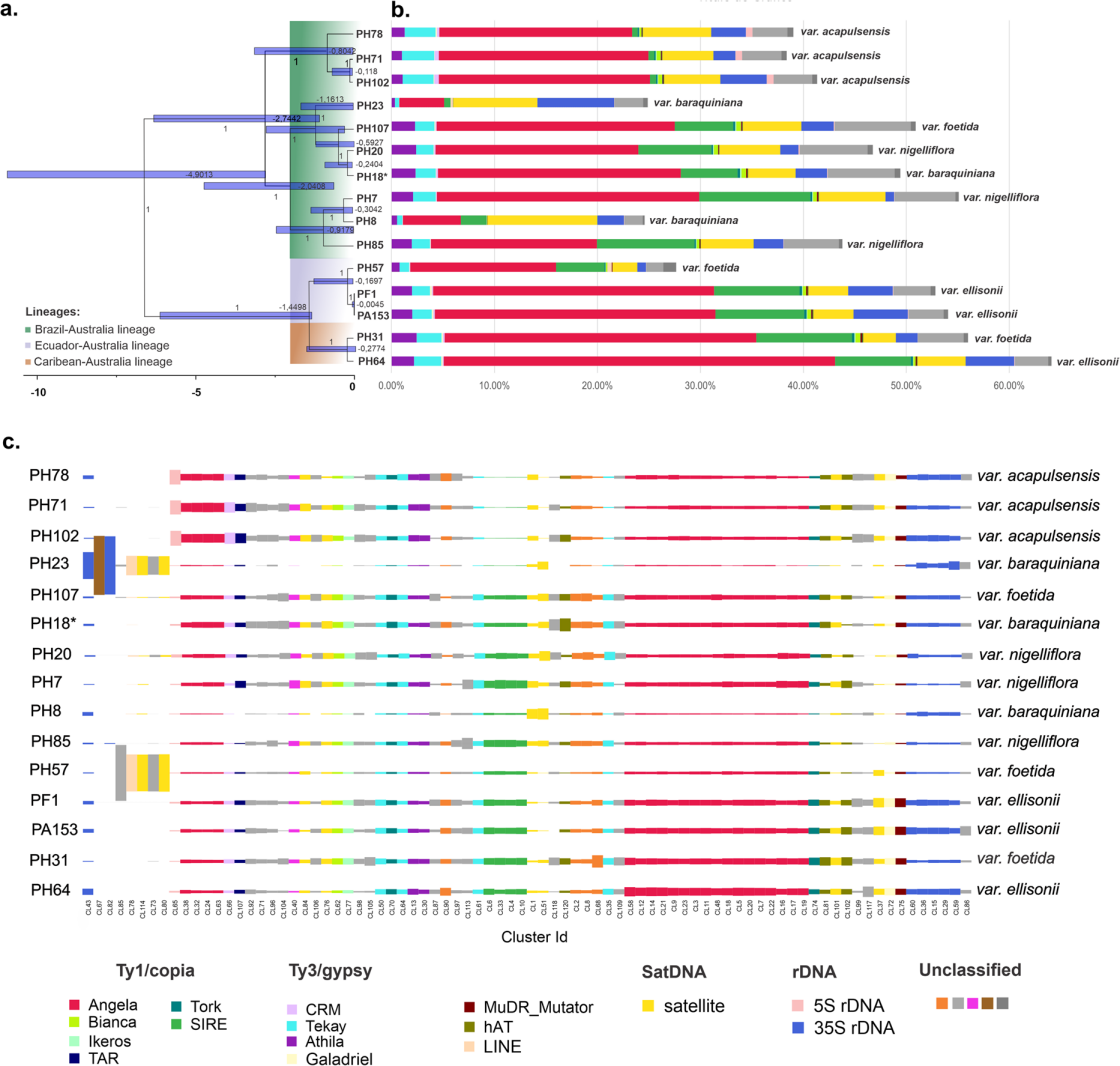
Comparative analysis of repetitive DNA abundance across different * Passiflora foetida* accessions representing distinct varieties. **a**. Phylogenetic relationships with divergence time estimates inferred from plastomes; **b**. Abundances per repeat class and lineage as percentage of the total genome size; **c.** Abundances per cluster proportional to the sizes of the bars, with accessions ordered according to phylogenetic relationships. Repeat annotations indicated by colours

The comparative analysis among different *P. foetida* accessions revealed a wide variation in the composition of the repetitive genome fraction, ranging from 24.58% to 64.10% (Table S4). *Passiflora foetida *var*. baraquiniana* and var. *foetida* showed considerable variation, with repetitive fractions spanning from 24.58% (PH8) to 49.42% (PH18) and from 27.65% in PH57 to 55.99% in PH31, respectively. *Passiflora foetida *var*. ellisonii* was characterized by the highest repetitive fractions, ranging from 52.83% (PF1) to 64.10% (PH64) (Fig. 2, Table 2).

Class I elements, particularly LTR retrotransposons, were the most representative repetitive components across all accessions. Within the Ty1/copia superfamily, the Angela lineage predominated in all varieties but exhibited marked variation in abundance, with the lowest values observed in var. *baraquiniana* (PH23–4.36%) and the highest in var. *ellisonii* (PH64–38%). The SIRE lineage also exhibited high variation among accessions, more than tenfold, reaching up to 10.79% in var. *nigelliflora* (PH7). Other repeat lineages were less abundant and contributed less to overall repeat fraction (Table S4).

Satellite DNAs also exhibited variation in abundance among populations, with the lowest values observed in once accession of var. *foetida* (PH31–3.17%) and the highest value in var. *baraquiniana* (PH8–10.65%; Table S4). Three identified satDNA families were previously described for other *Passiflora* species: two from *P. quadrangularis* (PquSat01-100 and PquSat06-1083), and one from *P. organensis* (PorSat04-1800), while two families were identified for the first time (Fig. S2). Satellite PfoSat01-27 was the only family which represented more than 1% of the genome, varying from 2.06 to 9.75% (4.7-fold), except for PfoSat02-31 in Pf023, in which it was the most abundant satDNA with 3.97%. PfoSat02-31 ranged from 0.11% to 3.97% in abundance (36-fold; Table 1). Variation in ribosomal DNA content was less pronounced, with the 35S rDNA most abundant in var. *baraquiniana* (PH23–8.34%) and least abundant in var. *nigelliflora* (PH7–1.27%). The 5S rDNA fraction was generally below 1% across all accessions (Table S4).

**Table 1:** Proportional abundance (%) of the main satellite DNAs identified in the comparative analysis among individuals of different *Passiflora foetida* varieties

Satellites	PfoSat01-27	PfoSat02-31	PquSat01-100	PquSat06-1083	PorSat04-1800
**var.**> ***acapulsensis***					
PH71	4.44%	0.15%	0.24%	0.05%	0.09%
PH78	5.56%	0.52%	0.49%	0.03%	0.03%
PH102	4.73%	0.13%	0.43%	0.04%	0.11%
**var.** ***baraquiniana***					
PH8	9.75%	0.54%	0.33%	0.01%	0.01%
PH18	3.80%	0.31%	0.32%	0.04%	0.14%
PH23	3.42%	3.97%	0.77%		0.01%
**var.** ***ellisonii***					
PF1	2.84%	0.50%	0.42%	0.02%	0.08%
PH64	3.15%	0.92%	0.57%	0.02%	0.03%
PA153	2.75%	0.55%	0.50%	0.01%	0.08%
**var.** ***foetida***					
PH31	2.66%	0.11%	0.28%	0.02%	0.09%
PH57	2.06%	0.15%	0.12%	0.02%	0.03%
PH107	4.89%	0.26%	0.39%	0.03%	0.11%
**var.** ***nigelliflora***					
PH7	6.10%	0.10%	0.13%	0.05%	0.09%
PH20	5.33%	0.21%	0.23%	0.02%	0.11%
PH85	4.64%	0.23%	0.17%	0.03%	0.06%

Except for var. *acapulsensis*, whose samples formed a monophyletic group and showed similar abundance profiles across the different repeat classes, the remaining varieties were not monophyletic. Relationships among accessions followed a predominantly geographic pattern (excepted for the non-native materials) based on both plastidial and nuclear DNA data (Figs. 2, S2 and S4). The repeatome variation observed within each variety was also evident when accessions were grouped into clades, suggesting that factors beyond phylogenetic relationships contribute to shaping repeat abundance. The most divergent accessions, with the lowest repeat abundances, were PH8, PH23, and PH57, which belonged to different clades and to two distinct varieties (Figs. 1, 2).

### Individual characterization of the repetitive fraction of *P. foetida*

To validate the repeatome characterization of *P. foetida*, an individual analysis was performed with the PH18 accession. The analysis revealed 86 repetitive clusters with an abundance greater than 0.01%, which corresponded to approximately 46% of the genome (Table 2), only slightly lower than observed for the same accession in the comparative analysis (~ 49%; Table S4). The Ty1/copia retrotransposon superfamily was the most abundant, representing 29.01% of the genome, with the Angela lineage alone accounting for 23.24%. The remaining Ty1/copia lineages were present at much lower abundances (0.06% to 5.45%), indicating a strong predominance of the Angela lineage within this superfamily. Satellite DNA accounted for 4.47% of the genome and was distributed across seven clusters, exceeding the proportion of Ty3/gypsy retrotransposons (3.34%), and highlighting the relatively low contribution of this superfamily in *P. foetida*. Within the Ty3/gypsy superfamily, the Chromovirus Tekay lineage was the most represented (1.47%), followed by Athila (1.31%) and CRM (0.05%). Ribosomal DNA sequences were present at lower proportions, with 5S and 35S rDNA representing 0.08% and 2.97% of the genome, respectively. The abundances of the individual lineages are similar to the ones observed in the comparative analysis. Additional individual analyses on the two most divergent accessions (PH23 and PH64 – Table S6) and on accession PF1, which was used for a whole genome assembly, also confirmed the contrasting repeat profiles observed in the comparative analysis (Table 2). Together, these results support that the observed differences reflect genuine variation among accessions.

**Table 2:** Repeatome characterization of *P. foetida* based on individual analyses performed with RepeatExplorer on 150 bp reads (accessions PH18, 23, 64 and PF1) or on whole genome assembly (accession PF1)

	*Passiflora foetida*				
Sequence source	Short reads	Short reads	Short reads	Short reads	Genome assembly
Acession	PH18	PH23	PH64	PF1	PF1
**Repeat**					
**Class I**					
**LTRs Ty3/gypsy**	3.34%		4.49%	3.24%	7.39%
Chromovirus					
CRM	0.05%		0.03%	0.02%	0.12%
Galadriel			0.06%	0.05%	0.22%
Reina					0.47%
Tekay	1.47%		1.68%	1.49%	4.10%
Non-chromovirus					
Athila	1.31%		2.73%	1.68%	2.41%
Ogre					
**LTRs Ty1/copia**	29.01%	1.90%	46.96%	38.61%	40.62%
Ale					0.16%
Alesia					0.02%
Angela	23.24%	1.87%	39.12%	29.55%	31.30%
Bianca	0.13%			0.08%	0.37%
Ikeros	0.06%		0.05%	0.03%	0.17%
Ivana					0.21%
SIRE	5.45%	0.03%	7.64%	8.95%	7.38%
TAR					0.18%
Tork	0.13%		0.14%		0.80%
**Unclassified LTRs**	5.66%	0.83%		3.20%	4.98%
LINEs		0.24%			7.17%
Pararetrovirus					0.05%
**Class II**					
hAT				0.01%	0.16%
CACTA					0.01%
MuDR_Mutator	0.02%		0.01%		0.01%
PIF_Harbinger					0.01%
**SatDNA**	4.47%	8.67%	4.51%	3.12%	1.82%
**rDNA**					
5S	0.08%	0.03%	0.09%	0.06%	
35S	2.97%	5.50%	4.42%	5.45%	0.41%
**Unclassified**	1.72%	3.73%	3.58%	3.41%	0.01%
**Total**	46.76%	20.89%	64.06%	57.10%	62.52%

*Genome size (1C) = 440,1 Mpb (Yotoko et al. 2011)

Six satDNA families were identified as tandem repeats in the individual analyses, one more (PfoSat03-447) than identified in the comparative analysis. PfoSat03-447 showed high confidence according to the TAREAN tool implemented in RepeatExplorer2, while three satDNAs showed low confidence, and two were identified based on sequence similarity with the *Passiflora* custom database and confirmed by sequence analyses (Table S5; Figs. S2a, S5). Two clusters (CLs 2 and 27) were similar to each other (85.2% identity—Fig. S2b) and were therefore considered to belong to the same satDNA family (PfoSat01-27). Three potentially species-specific satellite DNA families were identified: PfoSat01-27, a minisatellite (3.34% in abundance in PH18), PfoSat02-31 (0.31%), also a short repeat, with partial similarity to 35S rDNA, and PfoSat03-447, the only high-confidence cluster, with a monomer length of 447 bp and an abundance of 0.01% (Table S2; Fig. S5).

To confirm repeatome characterization from short reads, we also performed a detailed repeat characterization applying CARP to the available genome assembly of *P. foetida* PF1. Total repetitive fraction was ~ 62% in the whole-genome assembly, mostly because of a higher proportion of annotated LTR-retrotransposons. The assembled genome revealed higher abundances for all transposable elements, except for MuDR_Mutator, including 7.39% for the Ty3/gypsy superfamily, although this proportion remained considerably lower than that of Ty1/copia, which reached 40.62%. Tandem repeats, however, were possibly underestimated in the assembly (1.82%). In addition, the genome assembly enabled the detection of additional lineages within both superfamilies, reflecting the broader sampling of repetitive sequences provided by a largely complete, highly continuous genome. Nevertheless, total abundance was in general only slightly higher than estimated from clustering analysis of short reads (Table 2). Together, repeatome differences among accessions were confirmed, despite slight variation in proportions of annotated repeats using different approaches.

**Fig. 3:**
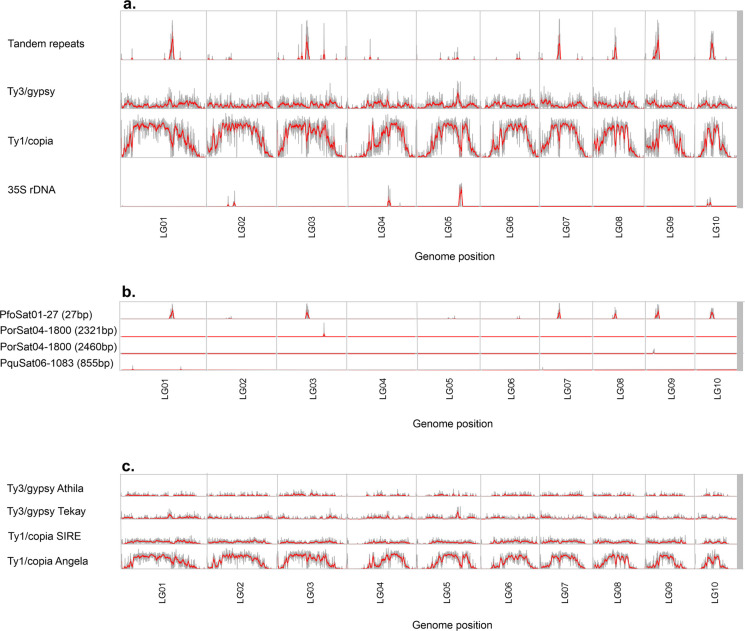
*In silico* distribution of repeats in the *P. foetida* genome. **a**. Overall distribution of the main repetitive DNA fractions in the genome. **b**. Main tandem repeats. **c**. Main LTR-RT lineages

*In silico* analysis of the repetitive fraction of the *P. foetida* genome assembly also enabled us to investigate the distribution of the different repeat lineages along the chromosomes. Ty1/copia superfamily was enriched in proximal chromosomal regions, with lower abundance at terminal regions, while Ty3/gypsy elements were less abundant and more dispersed along the entire chromosome length. Only three of the satellite families identified in the individual analysis of short reads were successfully mapped onto the *P. foetida* pseudomolecules, as well as the 35S rDNA, detected in four chromosome pairs, while the 5S rDNA was present in a single pair (Fig. 3a, S6).

The satellite PfoSat01-27 was detected in nine chromosomal pairs, with prominent peaks on pseudomolecules 1, 3, 7, 8, 9, and 10, whereas smaller or absence of peaks were observed on the remaining chromosomes, particularly pseudomolecule 4. Among the satellites shared with other species, PquSat06-1083, previously described for *P. quadrangularis*, was mainly detected on pseudomolecules 1 and 7, while PorSat04-1800, shared with *P. organensis*, was identified on pseudomolecules 3 and 9, both with monomers of different sizes when compared to the consensus of the other species (Fig. 3b).

The distribution of major repetitive lineages indicated that Ty3/gypsy Athila and Tekay elements were dispersed along the chromosomes but showed a tendency to accumulate along extended proximal chromosomal regions, particularly evident in LG10. A similar pattern was observed for Ty1/copia SIRE and Angela elements, with Angela being especially abundant in these regions, where peaks of enrichment are observed flaking the PfoSat01-27 peaks (Fig. 3c).

**Fig. 4:**
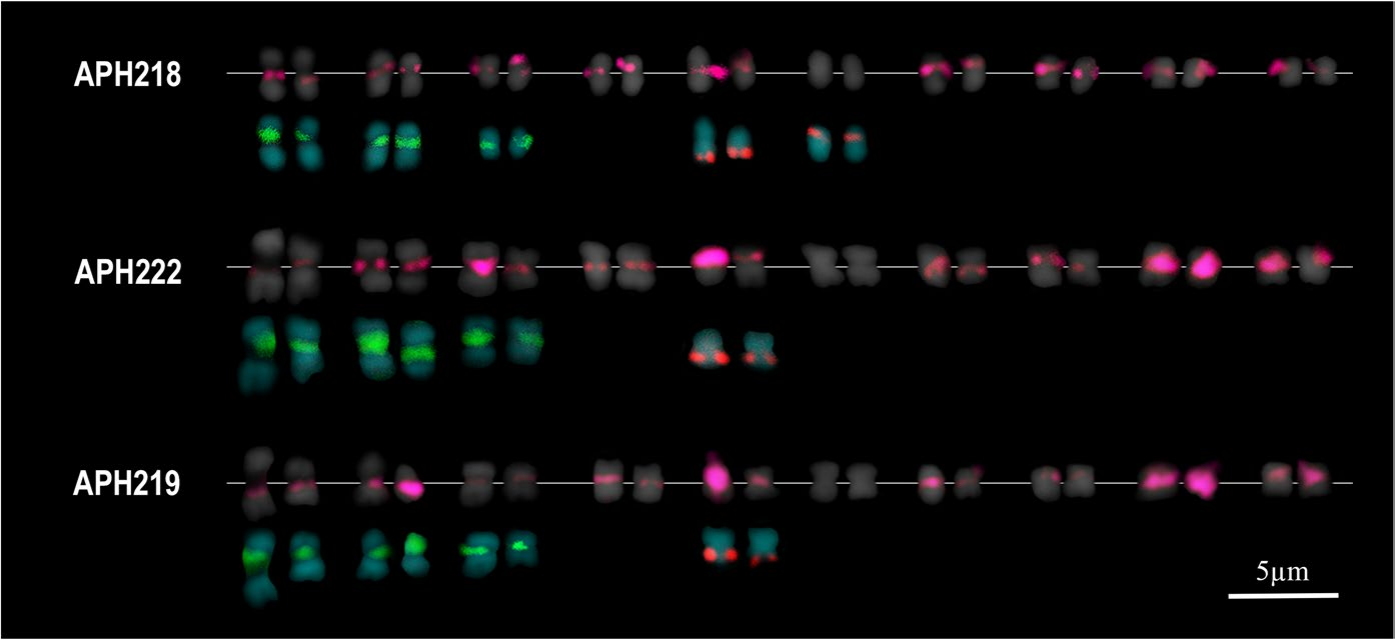
Karyogram showing the chromosomal distribution of the most abundant satellite of *Passiflora foetida*, PfoSat01-27 (pink), as well as the 35S (green) and 5S (red) rDNA. Heteromorphisms between homologous chromosomes in the abundance of satDNA signals could be detected using rDNA to identify some chromosome pairs

The most abundant satellites in *P. foetida* were further analysed by *in situ* hybridization (Fig. 4, S5). PfoSat01-27, identified as the most abundant tandem repeat, produced signals on nine chromosomal pairs across the analysed accessions, confirming the *in silico* results and showing a predominant (peri)centromeric localization. These signals were adjacent to, but not co-localized with the 35S rDNA loci, which were detected on three chromosomal pairs, one of them exhibiting weaker signal intensity. Variation among accessions was observed in the distribution of 5S rDNA, with one accession presenting an additional weak terminal pair of signals, whereas the others showed a single pair (Figs. 4, S7), consistent with the *in silico* genome analysis (Figure S4). Heteromorphic chromosomal pairs differing in signal intensity for both 5S rDNA and PfoSat01-27 were also detected (Figs. 4, S7).

Satellite DNA PquSat01-100 showed proximal blocks in *P. foetida* (Fig. S7), colocalized with the 35S rDNA (Fig. S6). In contrast, PquSat06-1083 exhibited a pericentromeric signal restricted to a single chromosome pair in the analysed accession (Fig. S7), while peaks on pseudochromosomes 1 and 7 (weaker) were observed in the genome assembly (Fig. S6).

### Comparative analysis among species of the subgenus *Passiflora*

To evaluate the extent of variation of *P. foetida* repeatomes in comparison to other species of the same subgenus, a comparative analysis was conducted including previously characterized species of the subgenus *Passiflora*: *P. cincinnata*, *P. edulis*, and *P. quadrangularis* (Sader et al. 2021), as well as species whose repeatomes were described here for the first time (*P. caerulea*, *P. coccinea*, *P. incarnata*, and *P. ligularis*). Additionally, two species from the subgenus *Decaloba* were selected as outgroups: *P. organensis* and *P. suberosa* (Table S6).

**Fig. 5:**
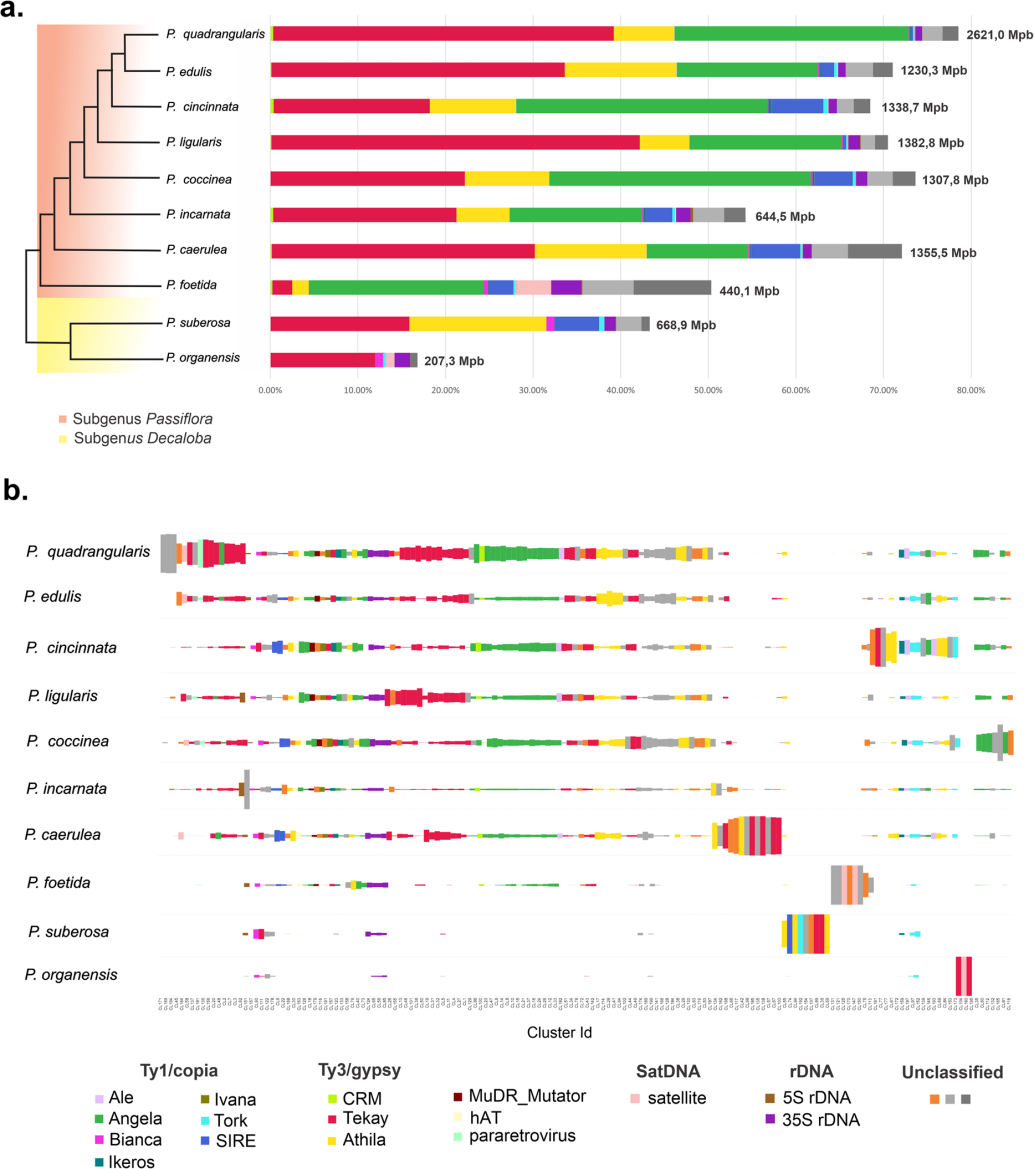
Comparative analysis of repetitive DNA in *Passiflora* genomes of the subgenera *Passiflora* and *Decaloba* (outgroup) represented according to their phylogenetic relationships inferred from plastomes. **a.** Abundances per repeat class and lineage as percentage of the total genome size; **b.** Abundances per cluster proportional to the sizes of the bars. Repeat annotations indicated by colours

The comparative analyses revealed a total of 199 clusters with an abundance higher than 0.01% across the analysed genomes, with the repetitive fraction ranging from 16.79% to 78.56% (Table S6), a substantial variation in repeat content across species. In this analysis, *P. foetida* PH18 showed the most divergent repeatome, in accordance with its phylogenetic position and it accounted for around ~ 50% of its genome, which was proportional to its known genome size in the context of this subgenus. Only *P. suberosa* showed a lower repeat abundance than expected considering its genome size, probably because it was the only polyploid species included and the increase in its genome size was due to whole genome duplication. Most of the variation was observed in the contribution of LTR retroelement superfamilies among species. The Ty1/copia was the most abundant not only in *P. foetida*, but also in *P. coccinea* and *P. cincinnata*, with predominance of Angela (Table S6, Fig. 5), but it was almost undetected in the *Decaloba* outgroup. In contrast, the Ty3/gypsy retrotransposon fraction was more abundant in the remaining species, ranging from 27.28% in *P. incarnata* to 47.81% in *P. ligularis*, including the *Decaloba* outgroup species (11.96% and 31.51%). Tekay was the most abundant lineage within this superfamily (up to 42.08%), followed by Athila, which was particularly prevalent in *P. caerulea* (12.75%) and *P. edulis* (12.76%). Class II elements were consistently present at low proportions across all analysed species (Table S6, Fig. 5).

Tandem repeat sequences exhibited substantial variation among species, with three satDNA families identified (PfoSat01-27, PquSat02-145 and PorSat01-161). PquSat02-145 and PorSat01-161 were identified as high-confidence satellites, while PfoSat01-27 appeared in two clusters and was classified as a low-confidence satellite. PquSat02-145 was the most widely shared among the analysed species and exhibited low sequence divergence, reaching its highest abundance in *P. edulis* (0.06%) and its lowest in *P. incarnata* (0.01%). *Passiflora foetida* showed the highest satDNA proportion (3.98%), while, in other species, satDNAs were present at low abundances, below 1%. The 35S rDNA was also most abundant in *P. foetida*, whereas *P. quadrangularis* exhibited the lowest proportion (0.8%). Although generally present at low levels, 5S rDNA reached its highest abundance in *P. incarnata* (0.24%; Table S6).

### Comparative analysis among species of section *Dysosmia*

The composition of the repeatome of *P. foetida* was also compared with two close relatives from the same section *Dysosmia* (*P. ciliata* and *P. vesicaria*). Again, the greatest absolute variation was observed in the abundance of the Angela lineage, which, although predominant in all three species, accounted for 17.41% in *P. ciliata*, to 28.66% in *P. vesicaria*. In contrast, SIRE lineage exhibited the highest relative variation, from 2.85% in *P. ciliata* to 10.17% in *P. vesicaria* (Table S7; Fig. S8). The largest divergence of *P. ciliata* was clearly observed in the comparison of individual repeat clusters, which did not align with the phylogenetic inference based on plastomes (Fig. S8). This pattern was reinforced by the divergence of five satDNA families, one of which was specific to *P. ciliata* (PclSat01-697; 0.84%; Fig. S2), while the others were shared among species, including the potentially centromeric satellite PfoSat01-27, which was most abundant in *P. ciliata* (2.95%; Table S5).

## Discussion

### *Passiflora foetida* and the subgenus* Passiflora*

The phylogenetic inference presented here corroborated *P. foetida* and section *Dysosmia* as sister to the remaining sections of the subgenus *Passiflora* (Cauz-Santos et al. 2020; Pacheco et al. 2020; Hopley et al. 2021; Sader et al. 2021), as well as the positions of *P. ligularis* and *P. incarnata*. However, the position of *P. coccinea* diverged from earlier results (Fig. S6), which may be related to the use of a larger dataset in the present analysis, but a lower species sampling.

In *P. foetida*, the Ty1/copia superfamily showed high abundance (29%), consistent with the pattern observed in most species of the subgenus *Passiflora* (Pamponét et al. 2019). This superfamily is mainly represented by the Angela and SIRE lineages, corroborating previous genome-based characterization (Zou et al. 2023). The predominance of Ty1/copia was also observed in *P. coccinea* and *P. cincinnata*, whereas the other analysed species exhibited a higher proportion of Ty3/gypsy elements. The Ty1/copia Angela and Ty3/gypsy Tekay lineages have been confirmed as the main contributors to genome size expansion in the genus, particularly within the subgenus *Passiflora* (Pamponét et al. 2019; Sader et al. 2021).

*Passiflora foetida* exhibited a very low abundance of Ty3/gypsy elements, especially Tekay (1.37%), which is likely associated with the small genome size of this species (Yotoko et al. 2011), the lowest among the genome sizes analysed here from the subgenus *Passiflora*, which ranged from 1 C = 440.1 Mbp in *P. foetida* to 2,621 Mbp in *P. quadrangularis*. Considering the higher proportion of Tekay elements in other genomes, including *P. organensis* with 1 C = 207.3 Mbp (Sader et al. 2021), and the phylogenetic position of *P. foetida*, our data may suggest either a lineage-specific elimination of Tekay in *P. foetida* (Ibarra-Laclette et al. 2013) or an independent amplification of this element in the subgenera *Passiflora* and *Decaloba*.

### Variation within *P. foetida*

The phylogenetic relationships among the *P. foetida* accessions sampled here were consistent with those reported by Hopley et al. (2021), who observed a predominant geographic pattern. The assessment of the repeatome in *P. foetida* at the intraspecific level, however, did not reflect its phylogenetic/geographic relationships. Furthermore, its repeatome was highly heterogeneous (24.58%–64.10%), comparable to that observed among species of the genus (16.79%–78.56%), which encompasses more than a ten-fold variation in DNA content (Sader et al. 2021). Variation in the abundance of the repetitive fraction is generally associated with variation in genome size in plant species (Albach and Greilhuber 2004; Novák et al. 2020). However, the current literature shows no marked evidence of genome size differences (1.29×) among *P. foetida* accessions or species complex (Table S8; Yotoko et al. 2011; Leite et al. 2019; Ferreira et al. 2020; Mikovski et al. 2021; Bugallo et al. 2023; Zou et al. 2023; Rolim et al. 2025; Zirpoli et al. 2025). The only accession used here for repeatome analysis for which a genome size estimation is available is the Chinese accession (referred here as PF1), whose genome was assembled and comprised 424.16 Mbp (Zou et al. 2023). We performed three estimations of its repeatome proportion (individual and comparative clustering of short reads and annotation of the repeats based on the final assemblies), with values ranging from 52.83%, from the comparative analysis to 62.52% based on the assembled genome. This variation based on different methodological approaches was lower than the intraspecific variation observed among *P. foetida* accessions, suggesting a so far undetected variation in genome size within this species complex. Considering the highest repeatome proportion observed for PH64 (~64%), PF1 value of 1 C = 424 Mb may be closer to the upper limit of the genome size variation of the species. Nevertheless, data on genome size and chromosome number are not available for the specific accessions whose repeatomes were analysed in the present study, limiting a more in-depth interpretation of the observed variation among these accessions, and indicating the need for further intraspecific investigation in *P. foetida*.

The repeatome of *P. foetida* is particularly diverse when compared to other species of the subgenus, suggesting a dynamic turnover of repeats. Although most individuals showed high abundance of Ty1/copia and scarcity of Ty3/gypsy, in two accessions (PH8 and PH23), for example, the proportion of tandem repeats exceeds that of Ty1/copia. Different species and even distinct populations of the same species may develop unique profiles of repetitive elements or variable patterns of sequences due to mutations and genomic rearrangements over time (Oliver et al. 2013; Ricci et al. 2018). This has been recently demonstrated, especially in pangenomic studies of species such as *Arabidopsis thaliana* L. (Kang et al. 2023; Lian et al. 2024) and *Solanum tuberosum* L. (Cheng et al. 2025). Divergence in the proportions of the repetitive fractions among *P. foetida* accessions, caused by fluctuations (amplification/deletion) of these elements, may be associated with environmental pressures or other factors yet to be investigated (Pons and Gillespie 2004; Feiner 2016; Castro et al. 2024). An alternative explanation for the significant variation observed is the potential hybrid origin of some *P. foetida* accession investigated with other species of the section *Dysosmia*. Indeed, hybridization between taxa within *Dysosmia* is known for co-occurring species in both native and non-native ranges (Vanderplank 2013; Hopley et al. 2026).

Phylogenetic analyses of the plastidial and nuclear datasets, together with repeatome characterization, did not support the subdivision of *P. foetida* into the currently accepted varieties. Nevertheless, the extensive intraspecific variation observed could be associated with reproductive isolation among accessions, impacting chromosomal pairing during meiosis or resulting in other incompatibilities (Levy 2013; Garrido-Ramos 2015; Castro et al. 2024). Recent studies indicate that repetitive DNA transposition may be influenced by the environment, often leading to the activation or silencing of genes (Kanazawa et al. 2009; Canapa et al. 2020; Castro et al. 2024). The different lineages of *P. foetida* require further characterization using an integrative approach, given that the morphological characters used by Vanderplanck (2013) to define varieties were shown to be homoplastic.

### Section *Dysosmia*

The phylogeny of section *Dysosmia* positioned *P. foetida* as sister to the remaining two species (*P. vesicaria* and *P. ciliata*), differing from the topology reported by Hopley et al. (2021), who investigated the same accessions under *P. foetida *sensu lato, and positioned them within the Caribbean-Australian lineage as sister accessions (Hopley et al. 2021). Both topologies are inconsistent with the greater divergence observed in the *P. ciliata* repeatome, especially in its satellitome, suggesting that the phylogenetic relationships and taxonomic status of species within this section should be re-evaluated with a larger sampling of this section (Vanderplank 2013; Parteka et al. 2021; Svoboda 2023). Despite the greater similarity between the clusters of *P. foetida* and *P. vesicaria* compared to those of *P. ciliata*, the differences in repetitive DNA abundance among these species are smaller than those observed among the varieties of *P. foetida*, suggesting that cryptic species might be present under the actual *P. foetida* species concept (Vanderplank 2013).

### The satellitome of* P. foetida *and related species from section *Dysosmia*

Among the analysed *Passiflora* species, the abundance of satDNA was highest in species from section *Dysosmia*. Four new satDNA were identified, which were amplified exclusively in *P. foetida* and closely related species. Among them, only PfoSat01-27 was detected in the broader comparative analysis, confirming its specificity In addition to the similarity with previously described satellites in the genus, the use of the *Passiflora* repeat database revealed low similarity between the 35S rDNA sequence and a new satDNA (PfoSat02-31). The same had been previously observed for PquSat01-100, from *P. quadrangularis*, which was hypothesized to have originated from a portion of its IGS region (Sader et al. 2021), suggesting multiple origins for satDNA in the genus from the 35S rDNA. The variability of 5S rDNA observed in this study has been previously reported in the group, with most accessions exhibiting a single chromosomal pair of sites (Zirpoli et al. 2025). And the heteromorphism in 5S rDNA sites has been documented among cultivars of *P. edulis* (Marróquín et al. 2023).

Satellite DNA typically forms blocks in the heterochromatic regions of chromosomes (Heslop-Harrison and Schwarzacher 2011; Ribeiro et al. 2017a, b). Satellite repeats may occur in subtelomeric or interstitial regions but are mostly found in centromeres (Garrido-Ramos 2015). The section-specific satDNA PfoSat01-27 hybridized to the (peri)centromeric region of nine chromosome pairs of *P. foetida*, which was confirmed *in silico*. Considering all satDNA localized so far in *Passiflora*, none had been typically centromeric (Costa et al. 2021; Sader et al. 2021). Although its association with centromeric proteins remains to be demonstrated, its chromosomal distribution suggests that PfoSat01-27 is a centromeric sequence, flanked mostly by Angela elements in the pericentromeres. As a putative centromeric satDNA, variability at the interspecific level is not uncommon (Macas et al. 2010; Melters et al. 2013; Zhang et al. 2013; Garrido-Ramos 2015). But the fact that it is section-specific and that its abundance varies widely among chromosomes of the species, even between homologous chromosomes generating heteromorphic pairs, suggests that this centromeric repeat is a recent evolutionary acquisition.

## Conclusion

Our results highlight the important role played by the repetitive fraction in the genome evolution of *P. foetida*, revealing high intra- and interspecific variation that might be associated with the presence of cryptic species or hybridization within session *Dysosmia*. The presence of satellite sequences shared with species from other subgenera supports its phylogenetic position; however, the intraspecific diversity of its repeatome, as well as the inconsistencies with phylogenetic and taxonomic data, reinforce the uncertain relationships among *P. foetida* varieties and closely related species. Finally, the identification of a potential centromeric satellite in *P. foetida* and related species suggests a recent evolutionary acquisition within section *Dysosmia*.

## Supplementary Information

Below is the link to the electronic supplementary material.

Supplementary file1 (DOCX 3.59 MB)

## Data Availability

All data generated or analysed during this study are available in GenBank (listed in the manuscript or in Supplementary Tables) or included in the manuscript.
