# Supplementary material for: A single species, different repeatomes? Genomic plasticity in Passiflora foetida L. complex and comparative insights across the subgenus Passiflora
Source: Chromosome Res. 2026 Aug 3;34(1):18. doi: 10.1007/s10577-026-09804-7 (PMC13429553; doi:10.1007/s10577-026-09804-7)
Supplement: Supplementary file 1 — Supplementary file1 (DOCX 3.59 MB) [file 10577_2026_9804_MOESM1_ESM.docx]

**A Single Species, Different Repeatomes? Genomic Plasticity in *Passiflora foetida* L. Complex and Comparative Insights Across the Subgenus *Passiflora***

*Arthur Monteiro^1^; Mariela A. Sader^2^; Jéssica Nascimento^3^; Gustavo Luna^1^; Daniela Cristina Imig^4^; Andrea Pedrosa Harand^1^*

**Supplementary Material**

**Supplementary Table 1.** Accessions of *Passiflora foetida* var. *foetida* used for *in situ* chromosomal analyses, collected directly in different Northeastern Brazilian states or obtained from germplasm banks

| **Accessions** | **Vouchers** | **Longitude** | **Latitude** | **Municipality** | **Source** |
| --- | --- | --- | --- | --- | --- |
| APH0100 | UFP90688 | -47.71 | -15.60 | - | Embrapa-DF |
| APH218 | UFP100030 | -34.83 | -7.10 | João Pessoa-PB | Field |
| APH222 | UFP100031 | -38.24 | -10.38 | Ribeira do Pombal-BA | Field |
| APH219 | - | -39.08 | -12.68 | - | Embrapa-BA |

**Supplementary Table 2.** Accessions from different *P. foetida* populations and their respective varieties used for comparative repeatome analysis. Status (native or non-native), geographic coordinates, collection country, and accession codes in the ENA or GSA platforms (PF1) are also presented. *Accessions also used for individual characterizations

|  | Status | Longitude | Latitude | Location | Code |
| --- | --- | --- | --- | --- | --- |
| ***Passiflora foetida*** |  |  |  |  |  |
| var. *acapulsensis* |  |  |  |  |  |
| PH71 | native | -97.2 | 18.1 | Mexico | ERR5419830 |
| PH78 | native | -86.30 | 13.16 | Nicaragua | ERR5419836 |
| PH102 | native | -95.95 | 16.54 | Mexico | ERR5419852 |
| var. *baraquiniana* |  |  |  |  |  |
| PH8 | native | -65.36 | -26.46 | Argentina | ERR5419787 |
| PH18* | native | -37.16 | -8.61 | Brazil | ERX5203483 |
| PH23* | native | -40.13 | -10.67 | Brazil | ERR5419797 |
| var. *ellisonii* |  |  |  |  |  |
| PF1* | non native | 114.30 | 22.36 | China | CRR889390 |
| PH64* | non native | 50.05 | -14.10 | Madagascar | ERR5419825 |
| PA153 | non native | 141.76 | -17.17 | Australia | ERR5419888 |
| var. *foetida* |  |  |  |  |  |
| PH31 | native | -71.56 | 18.39 | Dominican Republic | ERR5419803 |
| PH57 | native | -78.66 | -4.35 | Ecuador | ERR5419819 |
| PH107 | native | -34.83 | -7.09 | Brazil | ERR5419909 |
| var. *nigelliflora* |  |  |  |  |  |
| PH7 | native | -55.56 | -27.43 | Argentina | ERR5419786 |
| PH20 | native | -60.46 | 2.94 | Brazil | ERR5419795 |
| PH85 | nativa | -59.84 | -20.55 | Paraguai | ERR5419841 |

**Supplementary Table 3.** Accessions used for comparative analyses among species of the subgenus *Passiflora*, as well as for comparisons only within the section *Dysosmia* from subgenus *Passiflora*. Genome size data follow Melo & Guerra (2021), Yotoko et al. (2011), and Zirpoli et al., (2025). Only *P. ciliata* does not have genome size information available in the literature (-). Accessions codes for short read sequences from ENA or GenBank.are also provided.

|  | Genome size (1C) | Reference | Accession code |
| --- | --- | --- | --- |
| **Subg. Passiflora** |  |  |  |
| *P. caerulea* | 1355.5 Mpb | Yotoko et al. 2011 | ERR5419862 |
| *P. ciliata* | - | - | ERX5203540 |
| *P. cincinnata* | 1338.7 Mpb | Yotoko et al. 2011 | SRX9515500 |
| *P. coccinea* | 1307.8 Mpb | Yotoko et al. 2011 | ERR5419865 |
| *P. edulis* | 1230.3 Mpb | Yotoko et al. 2011 | ERR5419919 |
| ***P. foetida*** | 440.1 Mpb | Yotoko et al. 2011 | ERX5203483 |
| *P. incarnata* | 644.5 Mpb | Yotoko et al. 2011 | ERR5419921 |
| *P. liguralis* | 1382.8 Mpb | Yotoko et al. 2011 | ERR5419923 |
| *P. quadrangularis* | 2621.0 Mpb | Yotoko et al. 2011 | ERR5419924 |
| *P. versicaria* | 567.2 Mpb | Zirpoli et al. 2025 | ERX5203495 |
| **Subg. Decaloba** |  |  |  |
| *P. organensis* | 207.3 Mpb | Yotoko et al. 2011 | JAEPBF000000000 |
| *P. suberosa* | 668.9 Mpb | Yotoko et al. 2011 | ERX5203559 |

**Supplementary Table 4.** Comparative analysis of repeats from 15 accessions from five varieties of *P. foetida.* The genome size of 1C = 440.1 Mpb (Yotoko et al. 2011) was considered to calculate the resulting coverage (0.03x) after automatically read sampling (number of reads) during clustering analysis. Resulting proportions of reads in the major clusters corresponding to different repeats are indicated.

|  |  |  | var*. acapulsensis* | | | var*. baraquiniana* | | | var*. ellisonii* | | | var*. foetida* | | | var. *nigelliflora* | | |
| --- | --- | --- | --- | --- | --- | --- | --- | --- | --- | --- | --- | --- | --- | --- | --- | --- | --- |
|  |  |  | PH71 | PH78 | PH102 | PH8 | PH18 | PH23 | PF1 | PH64 | PA153 | PH31 | PH57 | PH107 | PH7 | PH20 | PH85 |
|  | Reads sampled |  | 105,972 | 106,390 | 106,114 | 106,458 | 106,772 | 106,010 | 107,598 | 105,776 | 106,998 | 106,756 | 105,864 | 106,124 | 107,138 | 106,314 | 106,140 |
| TEs |  |  |  |  |  |  |  |  |  |  |  |  |  |  |  |  |  |
| **Class I** | Ty3/gypsy |  | 4.59% | 4.64% | 4.60% | 1.11% | 4.51% | 0.76% | 4.06% | 5.08% | 4.20% | 5.15% | 1.82% | 4.40% | 4.41% | 4.28% | 3.82% |
| Chromovirus | | CRM | 0.41% | 0.24% | 0.45% | 0.02% | 0.17% | 0.02% | 0.14% | 0.10% | 0.14% | 0.16% | 0.07% | 0.15% | 0.09% | 0.14% | 0.03% |
|  |  | Galadriel | 0.04% | 0.13% | 0.08% | 0.02% | 0.05% | 0.00% | 0.17% | 0.14% | 0.16% | 0.13% | 0.03% | 0.07% | 0.03% | 0.05% | 0.05% |
|  |  | Tekay | 1.01% | 1.27% | 1.08% | 0.54% | 2.34% | 0.33% | 2.00% | 2.20% | 2.02% | 2.44% | 0.79% | 2.30% | 2.10% | 2.41% | 1.98% |
| Non-chromovirus | | Athila | 3.13% | 3.00% | 2.99% | 0.53% | 1.95% | 0.41% | 1.75% | 2.64% | 1.88% | 2.42% | 0.93% | 1.88% | 2.19% | 1.68% | 1.76% |
|  | Ty1/copia |  | 21.58% | 19.66% | 21.75% | 8.22% | 29.94% | 4.98% | 36.24% | 45.83% | 36.57% | 40.40% | 19.14% | 29.55% | 36.93% | 27.44% | 26.10% |
|  |  | Angela | 20.34% | 18.73% | 20.50% | 5.63% | 23.60% | 4.36% | 27.29% | 38.00% | 27.25% | 30.28% | 14.17% | 23.12% | 25.47% | 19.69% | 16.13% |
|  |  | Bianca | 0.42% | 0.19% | 0.41% | 0.07% | 0.46% | 0.03% | 0.33% | 0.20% | 0.37% | 0.48% | 0.14% | 0.46% | 0.37% | 0.41% | 0.29% |
|  |  | Ikeros | 0.08% | 0.05% | 0.06% | 0.01% | 0.12% |  | 0.09% | 0.06% | 0.08% | 0.11% | 0.03% | 0.10% | 0.09% | 0.09% | 0.07% |
|  |  | SIRE | 0.56% | 0.53% | 0.59% | 2.47% | 5.51% | 0.57% | 8.29% | 7.35% | 8.62% | 9.28% | 4.70% | 5.66% | 10.79% | 7.11% | 9.46% |
|  |  | TAR | 0.02% | 0.02% | 0.03% |  | 0.01% |  | 0.01% | 0.01% | 0.01% | 0.01% |  | 0.01% | 0.02% | 0.01% |  |
|  |  | Tork | 0.16% | 0.14% | 0.16% | 0.04% | 0.24% | 0.02% | 0.23% | 0.21% | 0.24% | 0.24% | 0.10% | 0.20% | 0.19% | 0.13% | 0.15% |
|  | LINEs |  |  |  |  |  |  | 0.23% |  |  |  |  | 0.44% | 0.01% |  | 0.01% |  |
| **Class II** |  |  |  |  |  |  |  |  |  |  |  |  |  |  |  |  |  |
|  |  | hAT | 0.07% | 0.07% | 0.07% | 0.01% | 0.10% | 0.00% | 0.08% | 0.07% | 0.08% | 0.13% | 0.03% | 0.08% | 0.07% | 0.05% | 0.03% |
|  |  | MuDR_ Mutator | 0.05% | 0.04% | 0.07% | 0.02% | 0.10% | 0.01% | 0.12% | 0.10% | 0.12% | 0.15% | 0.06% | 0.08% | 0.09% | 0.06% | 0.07% |
| **SatDNA** |  |  | 4.96% | 6.64% | 5.45% | 10.65% | 4.60% | 8.17% | 3.86% | 4.68% | 3.89% | 3.17% | 2.39% | 5.68% | 6.47% | 5.91% | 5.13% |
| **rDNA** |  | 5S | 0.63% | 0.66% | 0.67% | 0.03% | 0.07% | 0.03% | 0.07% | 0.07% | 0.05% | 0.04% | 0.03% | 0.07% | 0.05% | 0.14% | 0.04% |
|  |  | 35S | 3.18% | 4.85% | 4.88% | 3.47% | 3.29% | 8.48% | 4.98% | 5.63% | 5.69% | 2.37% | 1.47% | 3.56% | 1.27% | 2.31% | 1.84% |
|  | LTR Unclassified |  | 3.82% | 3.38% | 3.76% | 1.77% | 6.49% | 2.78% | 3.60% | 3.23% | 3.41% | 4.43% | 1.66% | 7.41% | 5.92% | 6.58% | 5.35% |
|  | Unclassified |  | 0.51% | 0.58% | 0.47% | 0.18% | 0.56% | 0.44% | 0.48% | 0.32% | 0.45% | 0.43% | 1.25% | 0.46% | 0.32% | 0.52% | 0.34% |
|  |  | **Total** | 38.38% | 39.02% | 41.34% | 24.58% | 49.42% | 24.88% | 52.83% | 64.10% | 54.06% | 55.99% | 27.65% | 50.90% | 55.10% | 46.75% | 43.80% |

**Supplementary Table 5.** Satellite DNAs identified in *Passiflora foetida* PH18 individual analysis, as well as in the comparative analyses within the subgenus *Passiflora*, and within the section *Dysosmia*. Cluster number, monomer size, GC content, and TAREAN confidence level are indicated for the individual analysis. “-” indicated satDNAs not detected by TAREAN but identified based on similarity to a custom database. Blank spaces indicate satDNA not detected in that sample

|  | PfoSat01-27 | PfoSat02-31 | PfoSat03-447 | PquSat01-100 | PquSat02-145 | PquSat06-1083 | PorSat01-161 | PorSat04-1800 | PclSat01-697 |
| --- | --- | --- | --- | --- | --- | --- | --- | --- | --- |
| ***P. foetida* individual analysis** | | | | | | | | | |
| Cluster | 2/28 | 31 | 107 | 34 |  | 75 |  | 70 |  |
| Monomer size (pb) | 27 | 55 | 447 | 1851 |  | 137 |  | 2487 |  |
| GC% | 50% | 77.4% | 38.5% | 50% |  | 37.2% |  | 29.7% |  |
| Confidence | Low | Low | High | Low |  | - |  | - |  |
| Abundance | 3.34% | 0.31% | 0.01% | 0.63% |  | 0.05% |  | 0.11% |  |
| **Comparative analyses** | | | | | | | | | |
| **Subg. *Passiflora*** |  |  |  |  |  |  |  |  |  |
| *P. caerulea* |  |  |  |  | 0.04% |  |  |  |  |
| *P. cincinnata* |  |  |  |  |  |  |  |  |  |
| *P. coccinea* |  |  |  |  | 0.03% |  |  |  |  |
| *P. edulis* |  |  |  |  | 0.06% |  |  |  |  |
| ***P. foetida*** | 3.98% |  |  |  |  |  |  |  |  |
| *P. incarnata* |  |  |  |  | 0.01% |  |  |  |  |
| *P. liguralis* |  |  |  |  |  |  |  |  |  |
| *P. quadrangularis* |  |  |  |  | 0.05% |  |  |  |  |
| **Subg. Decaloba** |  |  |  |  |  |  |  |  |  |
| *P. organensis* |  |  |  |  |  |  | 0.97% |  |  |
| *P. suberosa* |  |  |  |  |  |  |  |  |  |
| **Subg. *Passiflora* Section *Dysosmia*** |  |  |  |  |  |  |  |  |  |
| *P. ciliata* | 2.95% |  |  | 0.42% |  |  |  | 0.01% | 0.84% |
| ***P. foetida*** | **1.28%** |  |  | **0.36%** |  | **0.04%** |  | **0.11%** |  |
| *P. versicaria* | 1.11% |  |  | 0.21% |  | 0.02% |  | 0.10% |  |

**Supplementary Table 6.** Chromosome number (Melo & Guerra, 2021), 1C genome size in Mbp (Yotoko et al. 2011), number of sequences automatically sampled during the comparative clustering analysis (resulting in 0.03x coverage), and proportion of sequences in the major clusters corresponding to the repetitive fraction of eight species of the subgenus *Passiflora* and two species of the subgenus *Decaloba* identified by RepeatExplorer2.

|  |  |  | *P. cearulea* | *P. coccinea* | *P. cincinnata* | *P. quadrangularis* | *P. edulis* | *P. liguralis* | *P. incarnata* | ***P. foetida*** | *P. organensis* | *P. suberosa* |
| --- | --- | --- | --- | --- | --- | --- | --- | --- | --- | --- | --- | --- |
|  | *2n* |  | 18 | 18 | 18 | 18 | 18 | 18 | 18 | 20 | 12 | 24 |
|  | Genome size (1C) |  | 1355.5 | 1307.8 | 1338.7 | 2621.0 | 1230.3 | 1382.8 | 644.5 | 440.1 | 207.3 | 668.9 |
|  | Reads sampled |  | 466,296 | 450,010 | 477,950 | 902,074 | 423,924 | 476,722 | 221,998 | 151,300 | 71,520 | 230,588 |
| **TEs** |  |  |  |  |  |  |  |  |  |  |  |  |
| **Classe I** | Ty3/gypsy |  | 42.94% | 31.82% | 27.95% | 46.09% | 46.36% | 47.81% | 27.28% | 4.38% | 11.96% | 31.51% |
|  | Chromovirus | CRM | 0.18% | 0.02% | 0.36% | 0.32% | 0.11% | 0.11% | 0.33% | 0.24% |  |  |
|  |  | Tekay | 30.01% | 22.18% | 17.83% | 38.88% | 33.49% | 42.08% | 20.91% | 2.26% | 11.96% | 15.87% |
|  | Non-chromovirus | Athila | 12.75% | 9.62% | 9.76% | 6.89% | 12.76% | 5.62% | 6.04% | 1.88% |  | 15.64% |
|  | Ty1/copia |  | 17.76% | 35.95% | 35.71% | 27.42% | 18.33% | 18.11% | 18.89% | 23.55% | 0.87% | 6.63% |
|  |  | Ale | 0.04% | 0.03% | 0.11% | 0.07% | 0.04% | 0.04% | 0.03% |  |  |  |
|  |  | Angela | 11.57% | 29.85% | 28.64% | 26.73% | 16.14% | 17.39% | 15.12% | 19.97% | 0.01% |  |
|  |  | Bianca | 0.21% | 0.21% | 0.17% | 0.09% | 0.15% | 0.13% | 0.22% | 0.47% | 0.52% | 0.90% |
|  |  | Ikeros | 0.11% | 0.18% | 0.29% | 0.13% | 0.14% | 0.10% | 0.21% | 0.12% |  | 0.04% |
|  |  | Ivana | 0.01% | 0.02% | 0.02% | 0.01% | 0.01% | 0.01% | 0.03% |  |  |  |
|  |  | SIRE | 5.62% | 4.38% | 5.94% | 0.27% | 1.53% | 0.24% | 2.99% | 2.77% | 0.03% | 5.09% |
|  |  | Tork | 0.20% | 0.28% | 0.54% | 0.12% | 0.32% | 0.20% | 0.29% | 0.22% | 0.31% | 0.60% |
|  | Pararetrovirus |  |  | 0.01% |  | 0.05% | 0.01% | 0.01% | 0.01% | 0.01% |  |  |
| **Classe II** |  |  |  |  |  |  |  |  |  |  |  |  |
|  |  | hAT | 0.04% | 0.02% | 0.05% | 0.02% | 0.03% | 0.03% | 0.05% | 0.06% |  | 0.01% |
|  |  | MuDR_Mutator | 0.01% | 0.02% | 0.03% | 0.01% | 0.02% | 0.02% | 0.02% | 0.02% |  |  |
| **SatDNA** |  |  | 0.04% | 0.03% |  | 0.05% | 0.06% |  | 0.01% | 3.98% | 0.97% |  |
| **rDNA** |  | 5S | 0.01% | 0.01% | 0.01% | 0.01% | 0.03% | 0.08% | 0.29% | 0.11% | 0.01% | 0.04% |
|  |  | 35S | 1.00% | 1.29% | 0.93% | 0.80% | 0.87% | 1.34% | 1.65% | 3.46% | 1.79% | 1.74% |
|  | LTR Unclassified |  | 4.11% | 2.90% | 1.91% | 2.33% | 3.09% | 1.61% | 3.54% | 5.86% |  | 2.89% |
|  | Unclassified |  | 6.18% | 2.58% | 1.89% | 1.81% | 2.25% | 1.46% | 2.44% | 8.87% | 0.82% | 0.95% |
|  |  | **Total** | 72.11% | 73.64% | 68.48% | 78.56% | 71.05% | 70.49% | 54.24% | 50.35% | 16.79% | 43.31% |

.

**Supplementary Table 7.** Proportion of the genome (%) composed of repetitive sequences identified in the comparative RepeatExplorer2 analysis of three species from the *Dysosmia* section of the subgenus *Passiflora*

|  | ***P. ciliata*** | ***P. foetida*** | ***P. vesicaria*** |
| --- | --- | --- | --- |
| 2*n* | - | 20 | 20 |
| Genome size (1C) | - | 440.1 Mbp | 567.2 Mpb |
| Sampled reads | 439,052 | 437,036 | 438,968 |
| Resulting coverage | - | 0.1x | 0.1x |
| ***Repeat*** |  |  |  |
| **Class I** |  |  |  |
| LTR Ty3/gypsy | 0.77% | 2.67% | 2.36% |
| Chromovirus |  |  |  |
| CRM | 0.03% | 0.20% | 0.17% |
| Galadriel |  | 0.01% | 0.02% |
| Tekay | 0.74% | 2.46% | 2.17% |
| Non-Chromovirus |  |  |  |
| Athila | 0.94% | 2.09% | 2.61% |
| LTR Ty1/copia | 21.30% | 30.78% | 42.15% |
| Angela | 17.41% | 22.41% | 28.66% |
| Bianca | 0.05% | 0.52% | 0.42% |
| Ikeros | 0.02% | 0.13% | 0.11% |
| SIRE | 2.85% | 5.42% | 10.17% |
| TAR |  | 0.04% | 0.04% |
| Tork | 0.03% | 0.17% | 0.14% |
| Unclassified LTRs | 1.10% | 6.33% | 3.94% |
| LINEs | 0.65% |  |  |
| **Class II** |  |  |  |
| MuDR_Mutator | 0.01% | 0.06% | 0.05% |
| hAT | 0.01% | 0.06% | 0.05% |
| **SatDNA** | 4.22% | 1.79% | 1.43% |
| **rDNA** |  |  |  |
| 5S | 0.05% | 0.09% | 0.04% |
| 35S | 2.45% | 5.95% | 2.03% |
| **Unclassified** | 17.69% | 3.58% | 2.31% |
| **Total** | 51.30% | 48.14% | 54.06% |

**Supplementary Table 8.** Reported genome size estimates for *P. foetida* obtained from different studies. The table summarizes 2C and 1C DNA content values in pg, together with their respective references and DOI numbers, highlighting the variation in genome size estimates reported for the species across independent analyses.

| ***Passiflora foetida*** |  |  |  |
| --- | --- | --- | --- |
| **2C** | **1C** | **Reference** | **DOI** |
| 0.962 | 0.48 | Yotoko et al. 2011 | [10.1371/journal.pone.0018212](https://doi.org/10.1371/journal.pone.0018212) |
| 1.04 | 0.52 | Leite et al. 2019 | [10.1007/s11240-018-01536-9](https://doi.org/10.1007/s11240-018-01536-9) |
| 1.04 | 0.52 | Ferreira et al. 2020 | [10.1016/j.scienta.2020.109532](https://doi.org/10.1016/j.scienta.2020.109532) |
| 1.18 | 0.59 | Mikosvski et al. 2021 | [10.1007/s11240-021-02120-4](https://doi.org/10.1007/s11240-021-02120-4) |
| 0.96 | 0.48 | Bugallo et al. 2023 | [10.1590/2175-7860202374046](https://doi.org/10.1590/2175-7860202374046) |
| 0.912 | 0.456 | Zou et al. 2023 | [10.1186/s43897-023-00076-x](https://doi.org/10.1186/s43897-023-00076-x.) |
| 1.12 | 0.55 | Rolim et al. 2025 | [10.1590/1413-7054202549014124](https://doi.org/10.1590/1413-7054202549014124) |
| 1.07 | 0.535 | Zirpolo et al. 2025 | 10.1080/14772000.2025.2565219 |


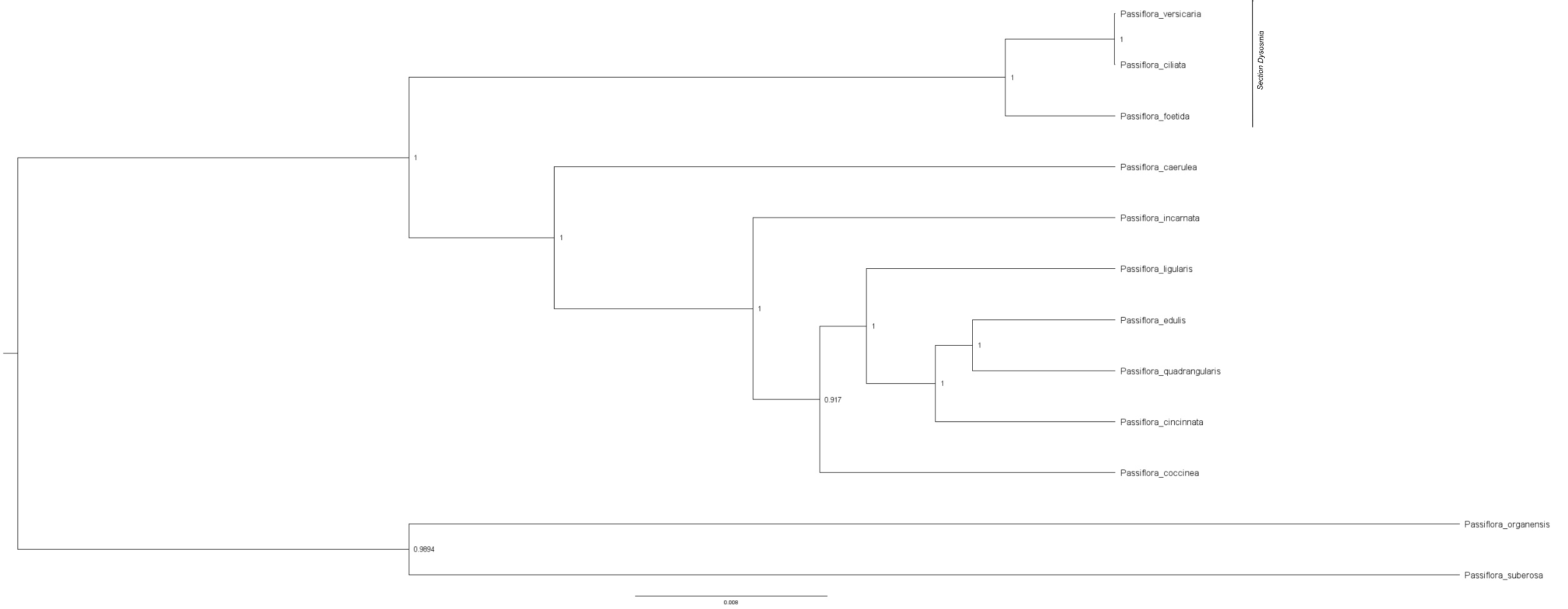


**Supplementary Figure 1.** Plastome phylogeny of the subgenus *Passiflora*, with the subgenus *Decaloba* used as the outgroup. The bar highlights the section *Dysosmia*,which is also included within the subgenus *Passiflora*

**
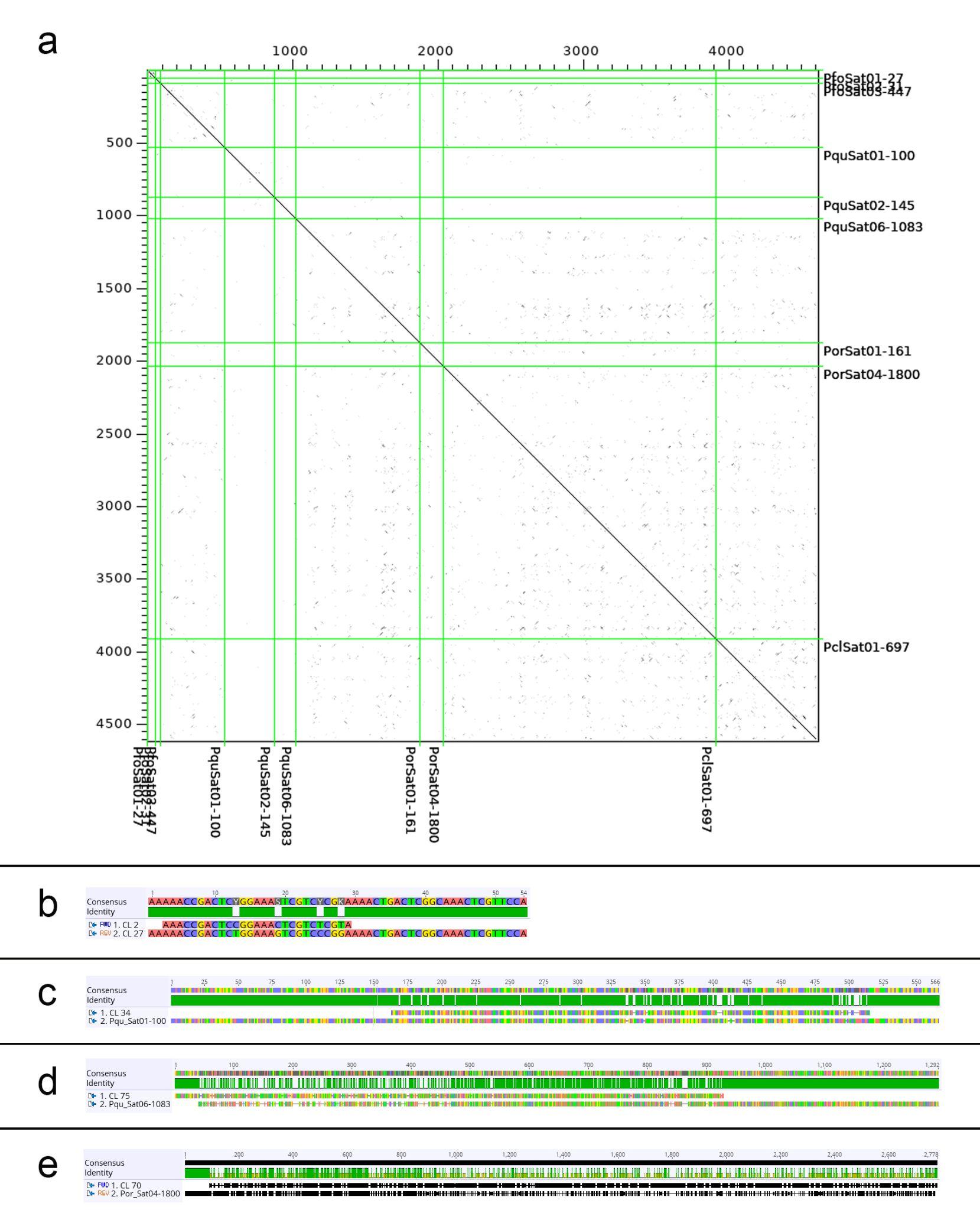
**

**Supplementary Figure 2.** Dot plot generated in Dotter and pairwise alignments performed in Geneious Prime using the Global alignment with free end gaps algorithm. a) Dot plot generated with satellite DNA sequences of *Passiflora foetida* (Pfo) and from other species (*P. quadrangularis* - Pqu, *P. organensis* - Por, and *P. caerulea* - Pcl) showing similarity in the repeatome database analysis. b) Pairwise alignment between PfoSat1-27 clusters CL2 (27 bp) and CL27 (54 bp), showing 85.2% sequence similarity. c) Pairwise alignment between Pfo CL34 (339 bp) and PquSat01-100 (558 bp), showing 87% sequence similarity. d) Pairwise alignment between Pfo CL75 (860 bp) and PquSat06-1083 (1083 bp), showing 66.3% sequence similarity. e) Pairwise alignment between Pfo CL70 (2478 bp) and PorSat04-1800 (1800 bp), showing 52.3% sequence similarity.


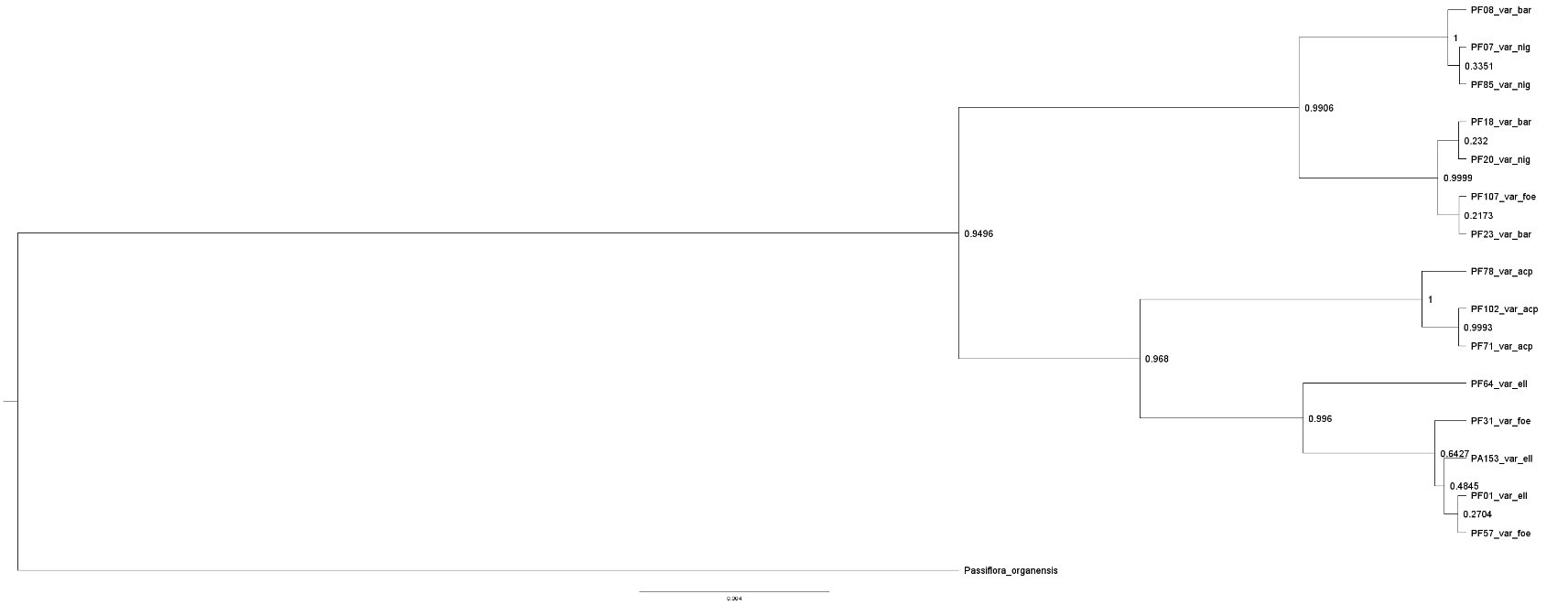


**Supplementary Figure 3.** Phylogenetic relationships inferred from nuclear assemblies of *Passiflora foetida* accessions.


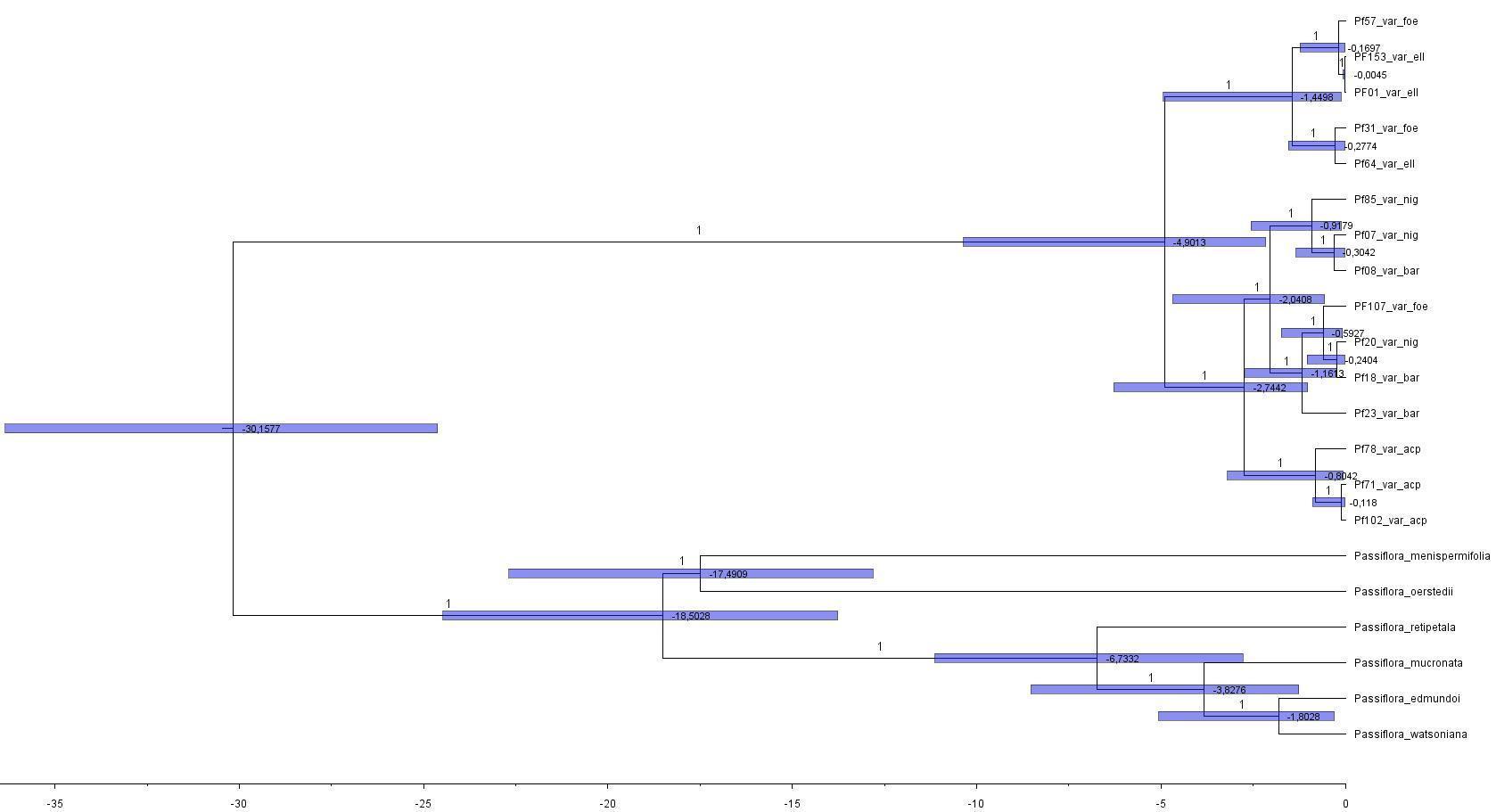


**Supplementary Figure 4.** Phylogenetic relationships inferred from plastome assemblies using *P. foetida* as a reference, with divergence time estimates based on fossil records of *Passiflora.*


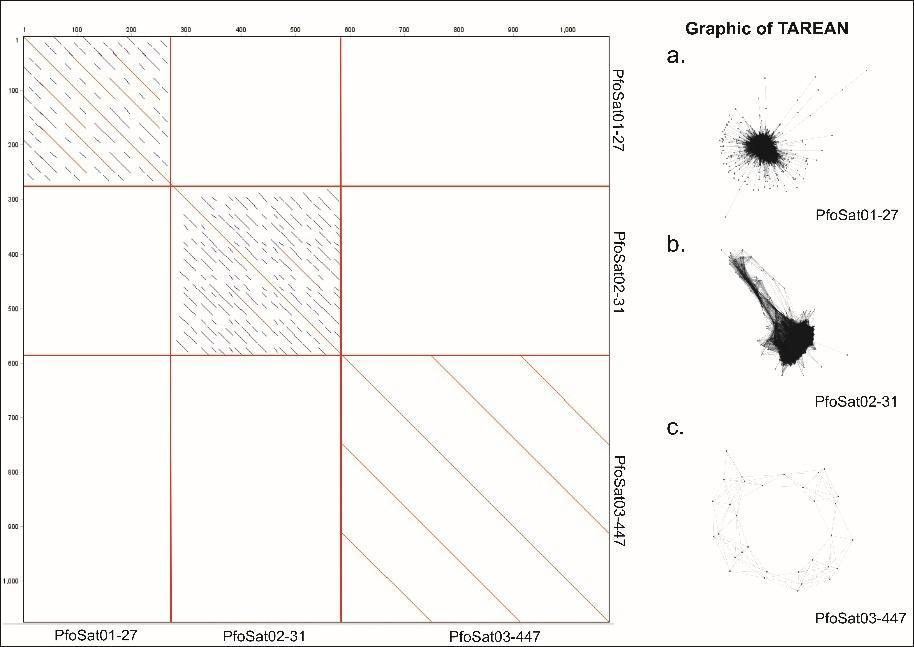


**Supplementary Figure 5.** Organization of *P. foetida* satellite DNAs. Left, dotplot of contigs generated in Geneious showing tandem repetitions, and, right, corresponding graphs generated by TAREAN for: a. PfoSat01-27; b. PfoSat02-31; and c. PfoSat03-447.

**
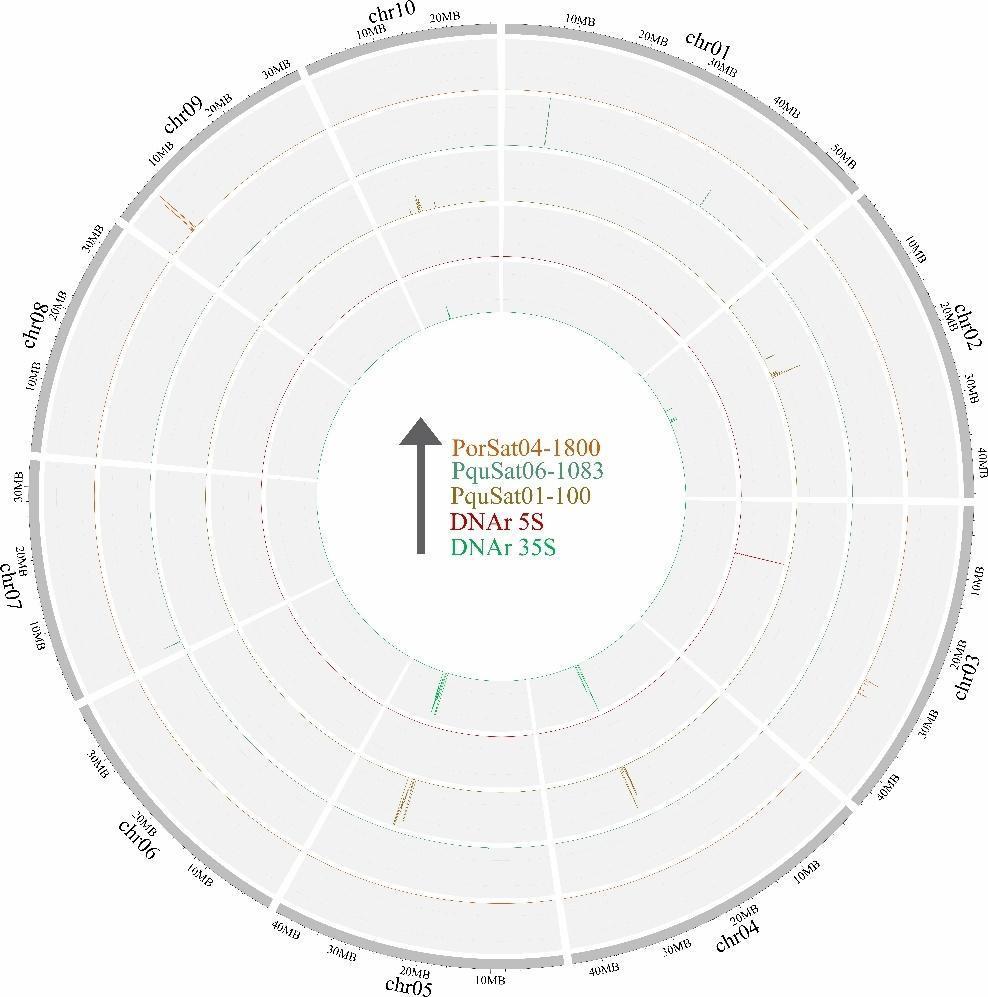
**

**Supplementary Figure 6.** Distribution of shared satellites DNAs (PquSat01-100; PquSat06-1083; PorSat04-1800), as well as 5S and 35S rDNA, in *P. foetida* pseudochromosomes. The arrow in the centre indicates the order of the sequences, from the centre to the periphery.

**
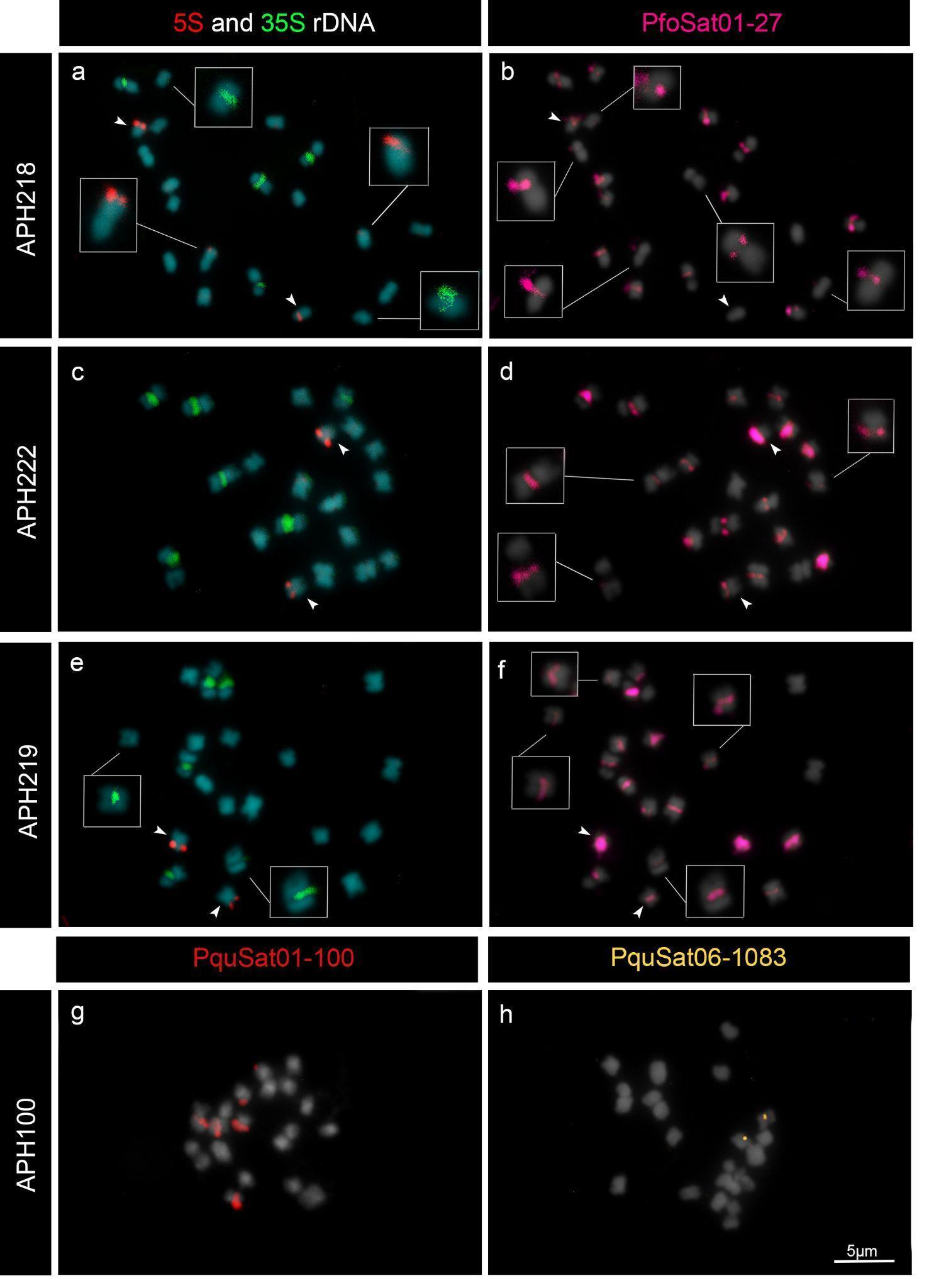
**

**Supplementary Figure 7.** *In situ* localization of tandem repeats in *Passiflora foetida* metaphase chromosomes. 5S rDNA (red) and 35S rDNA (green) in a, c, e. Species-specific satellite PfoSat01-27 (pink) in different *P. foetida* accessions in b, d, f. PquSat01-100 (red) in g. PquSat06-1083 (yellow) in h. Insets show weaker signals in higher brightness. Arrowheads point to heteromorphic chromosome pairs.

*
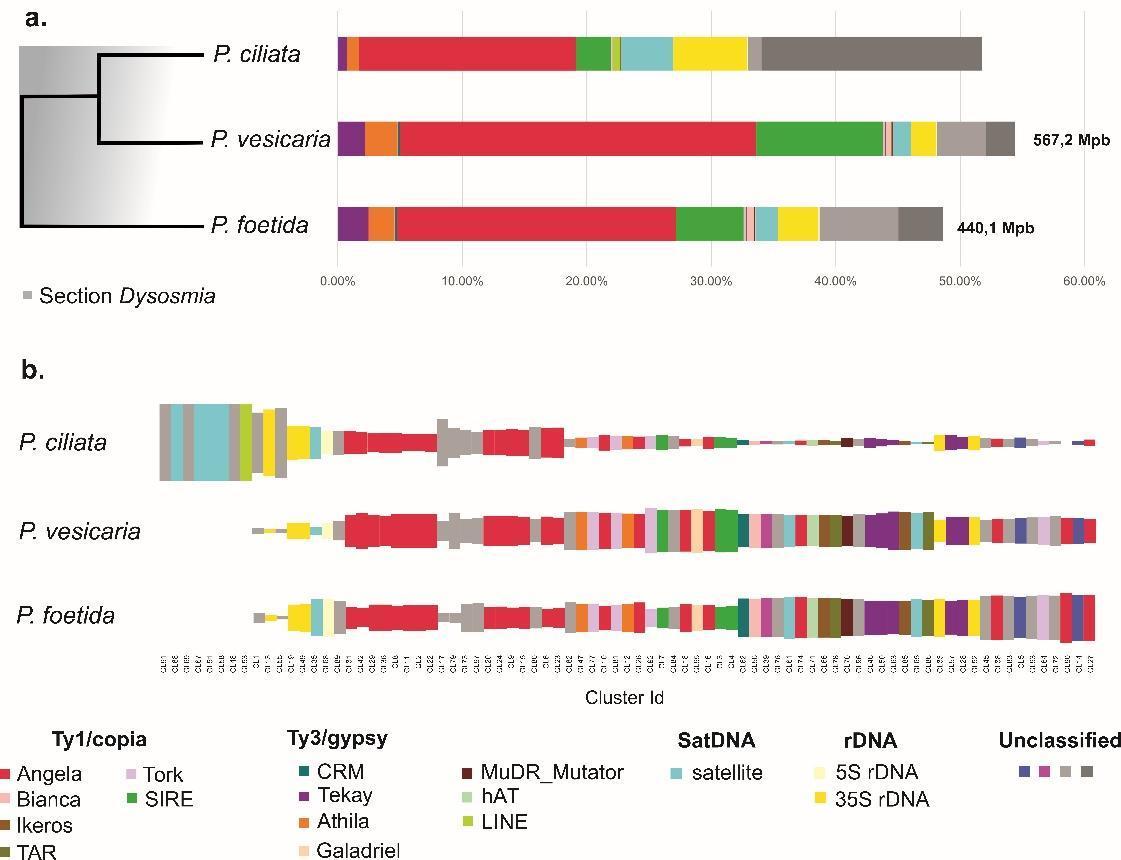
*

**Supplementary Figure 8.** Comparative abundance of repetitive DNA in different species of the section *Dysosmia*. **a.** Phylogenetic relationships inferred from plastome assemblies using *P. foetida* as a reference, with divergence time estimates based on fossil records of *Passiflora*; **b.** Abundances per repeat class and lineage; **c.** Abundances per cluster with annotations indicated by colours**.**
